# Transcriptional feedback in the insulin signalling pathway modulates ageing in both *Caenorhabditis elegans* and *Drosophila melanogaster* †
†Electronic supplementary information (ESI) available. See DOI: 10.1039/c3mb25485bClick here for additional data file.



**DOI:** 10.1039/c3mb25485b

**Published:** 2013-04-26

**Authors:** Dobril K. Ivanov, Irene Papatheodorou, Matthias Ziehm, Janet M. Thornton

**Affiliations:** a EMBL-European Bioinformatics Institute (EBI) , Wellcome Trust Genome Campus , Hinxton , Cambridge , CB10 1SD , UK . Email: divanov@ebi.ac.uk; b Institute of Healthy Ageing , Department of Genetics Evolution and Environment , University College London , Darwin Building , Gower Street , London , WC1E 6BT , UK

## Abstract

Several components have been previously identified, that modulate longevity in several species, including the target of rapamycin (TOR) and the Insulin/IGF-1 (IIS) signalling pathways. In order to infer paths and transcriptional feedback loops that are likely to modulate ageing, we manually built a comprehensive and computationally efficient signalling network model of the IIS and TOR pathways in worms. The core insulin transduction is signalling from the sole insulin receptor *daf-2* to ultimately inhibit the translocation of the transcription factor *daf-16* into the nucleus. Reduction in this core signalling is thought to increase longevity in several species. In addition to this core insulin signalling, we have also recorded in our worm model the transcription factors *skn-1* and *hif-1*, those are also thought to modulate ageing in a *daf-16* independent manner. Several paths that are likely to modulate ageing were inferred *via* a web-based service NetEffects, by utilising perturbed components (*rheb-1*, *let-363*, *aak-2*, *daf-2*;*daf-16* and *InR*;*foxo* in worms and flies respectively) from freely available gene expression microarrays. These included “routes” from TOR pathway to transcription factors *daf-16*, *skn-1*, *hif-1* and *daf-16* independent paths *via skn-1*/*hif-1*. Paths that could be tested by experimental hypotheses, with respect to relative contribution to longevity, are also discussed. Direct comparison of the IIS and TOR pathways in both worm and fly suggest a remarkable similarity. While similarities in the paths that could modulate ageing in both organisms were noted, differences are also discussed. This approach can also be extended to other pathways and processes.

## Abstract 2

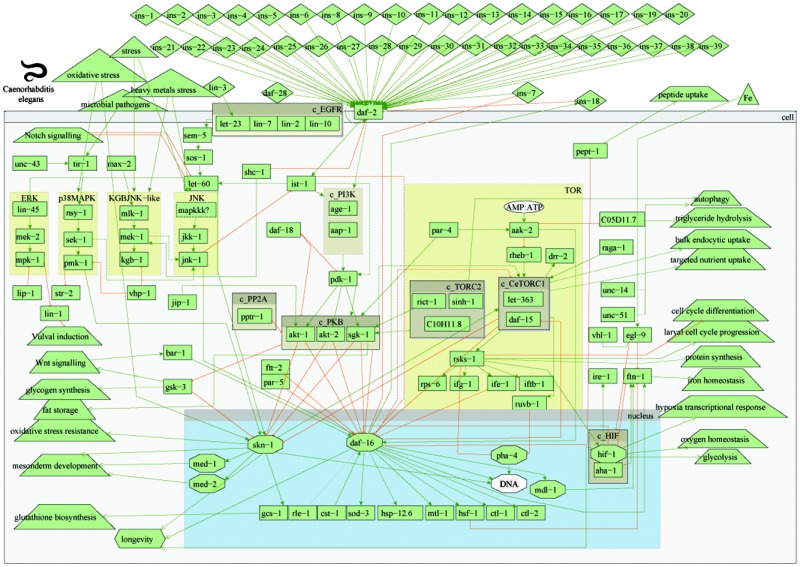
Using manually curated signalling models of the IIS/TOR pathways, transcriptional feedback is shown to modulate ageing in worms and flies.

## 1. Introduction

The ageing process, defined as a decrease in the ability of organisms to respond to environmental stimuli, stress, decline in physiological functions and inevitable death, is a pliable biological process. Interventions, such as dietary restriction (DR, reduction in calorie intake without malnutrition), have been previously shown to robustly extend lifespan in a range of species, from yeast to mammals and possibly primates.^1^ Inevitably, there is a general interest in interventions and mechanisms that underlie or affect longevity in humans, despite the restrictions on experimental interventions or genetic manipulations. Because of long life expectancy, the use of primates as a proxy to humans, to investigate potential mechanisms of ageing is a lengthy process.^1^ However, some of the mechanisms are conserved over large evolutionary distances^2^ and therefore the use of short-lived model organisms is a fruitful and convenient choice.

Several components and biological pathways have been identified that modulate the ageing process, including nutrient sensing pathways, the target of rapamycin (TOR)^3,4^ and the insulin/insulin-like growth factor (IIS) signalling.^5^ Moreover, the first pathway discovered which can regulate ageing is the IIS pathway in the roundworm *Caenorhabditis elegans* (reviewed in Kenyon *et al.* 2011^6^). Following these observations, genetic variants in human orthologues of several worm IIS components, among others the forkhead box class O transcription factor (FOXO, *daf-16* in worms), have been found to be associated with exceptional longevity in genome-wide association studies in humans (for a list of genes and studies see Kenyon *et al.* 2011^6^).

One of the most widely used techniques to study the role of component proteins in a biological pathway in model organisms is the overexpression or knockout of specific genes, followed by experimental determination of the effects that such perturbations cause. Numerous perturbations in the IIS signalling pathway have been reported to affect lifespan in several species. In worms and flies these include, along with many others, the insulin receptor *InR* ^7^/*daf-2*,^8,9^ *chico*,^10^ *skn-1*,^11^ *hif-1*,^11^ Pi3K/*age-1*,^12^ *foxo*/*daf-16*,^13,14^ *pha-4* ^15^ and several components of the TOR pathway.^16^ The insulin signalling pathway is a neuroendocrine pathway that among other functions monitors nutrients on the whole organism level.^17^ Similarly, the TOR pathway is also a nutrient-sensing pathway, although it monitors the levels of intracellular nutrients.

The basic observation in multiple organisms is that a reduction in insulin signalling activity (either by interfering with components of the pathway or partly by DR) extends lifespan. This process is thought to proceed by increasing the translocation of the FOXO transcription factor (*daf-16* and *foxo* in worms and flies) into the nucleus. In turn, FOXO modulates the expression of a multitude of genes^18^ and the side effect is extended lifespan. So, predicting the effects of gene interference is very complex.

Following several lines of evidence, multiple signals that affect the nutrient sensing properties within the IIS and TOR pathways modulate ageing. In this paper we have made an attempt to capture the current knowledge of the IIS and TOR pathways in worm and how their modulation affects longevity, in data that we have collected in a consistent manner. This gave the ability to programmatically explore and differentiate between potential signalling “routes” within the IIS and TOR pathways that ultimately lead to changes in longevity. We utilised publicly available genome-wide gene expression microarray experiments that perturb genes within the IIS and TOR pathways. We also made inferences about paths, including transcriptional feedback loops, which support or contradict the observed ageing phenotype (short and long-lived) in these experiments. Furthermore, we have attempted a direct comparison between the effects of perturbation of orthologous genes in the worm and fly with respect to longevity phenotype recorded in both organisms. These experiments comprised *daf-2*;*daf-16*, *rheb-1*, *let-363*, *aak-2* in worms and *InR*;*foxo* in *Drosophila*. Using our model and these publicly available microarray studies we are able to draw inferences with respect to several functionally important paths within the IIS and TOR pathways. These included paths to longevity *via skn-1*, *daf-16*/*foxo* and *hif-1*.

## 2. Materials and methods

### 2.1. A signalling network model of the insulin and TOR pathways in *C*. *elegans*

In order to explore “routes” or paths within the IIS and TOR pathways that could potentially modulate longevity, a comprehensive knowledge of the genes part of the two pathways and their connectivity was required. Such knowledge, not only needed to be organised in a computationally efficient way of representing the different components, but also had to be visually portrayed in a manner that humans can understand. Thus, we built a signalling network model of the IIS and TOR pathways by using GraphML, a modified version of the Extensible Markup Language (XML), supported in the program yEd (http://www.yworks.com). These allowed the required formal interpretation and at the same time a graphical representation of the pathways.

Building the model, *i.e.* genes and their interactions/connections, of the two pathways required extensive literature review of the existing knowledge by utilising at first several review articles.^5,6,19–22^ In addition, primary sources based on PubMed literature searches (; http://www.ncbi.nlm.nih.gov/pubmed/), utilising a permutations of the insulin and TOR pathways names and gene symbols already recorded in the model, were also followed. Evidence suggesting components and their interactions were always followed to their primary source and interactions between components that were only suggestive or hypothetical were omitted. Thus, only connections that were shown as derived from experimental evidence were included. Protein–protein interactions, based on yeast two-hybrid system or other screens were also not included, due to the lack of directionality of the connections.

**Fig. 1:**
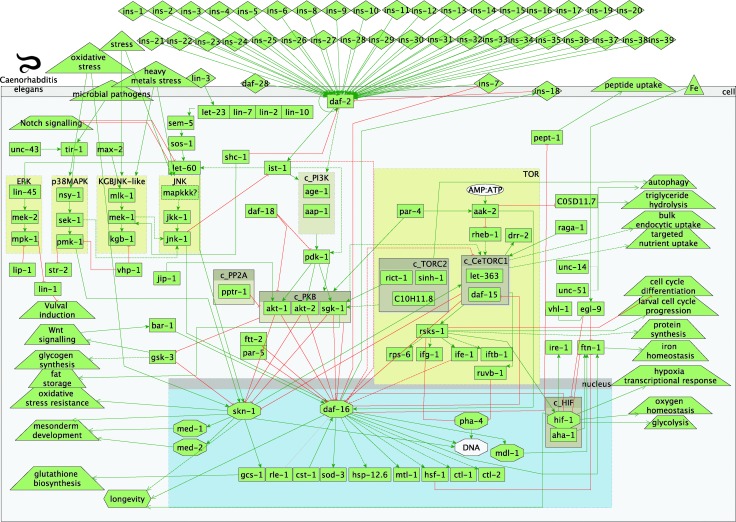
Overview of worm insulin and TOR pathways. Legend: rectangles represent genes; diamonds – molecules; triangles – environmental effects; trapezoids – other than IIS or TOR pathways; octagons – transcription factors; green arrow lines represent activation; red t-shaped lines represent inhibition; brown boxes starting with c_ represent complexes.

An overview of the model of the worm IIS and TOR signalling pathways in *C. elegans* and their interactions is presented in Fig. 1. The graph comprises activation and inhibition relationships between the protein components of the signalling network in the IIS and TOR pathways. A detailed description of the signalling is given in 3.1. In addition, all gene names and associated WormBase/Ensembl identifiers, part of the model of IIS and TOR pathways in worms, are listed in Table S1 (ESI†). All references used to build the worm connections/interactions within the TOR and IIS pathways are listed in Table S2 (ESI†).

### 2.2. Whole-genome expression datasets

Having built a comprehensive model of the IIS and TOR pathways, we wanted to explore potential transcriptional feedback and paths that could modulate longevity. The most widely used technique that provides information on the effect of components in biological pathways is gene knockout or overexpression of specific genes. This is followed by microarray gene expression experiments to study the effect of these perturbations. For this reason all gene names and symbols, part of the two worm pathways, were used to search for available experiments in ArrayExpress^23^ and GEO^24^ databases. These databases are a comprehensive source of functional genomics experiments, including gene expression studies. Since microarray experiments are the most widely used technique to study the effect of genes in a particular biological pathway, we concentrated on microarray gene expression studies to try to maximise available experiments. In order to achieve consistent gene expression profiles across experiments and across species, data on such experiments needed to be in raw format to allow unified data analysis. Moreover, only experiments that had recorded longevity as phenotype were considered.

As a result, only four experiments were found during these searches. In total there were three worm studies comprising four whole worm microarray expression profiles and one whole fly experiment. Visual representation of the perturbed components can be found in Fig. 3. The identifiers, perturbed genes, references and phenotype outcome can be found in Table 1. The four worm microarray experiments comprised RNAi (i stands for double-stranded RNA interference) inhibition of the *rheb-1* and *let-363* genes, part of the TOR pathway, and loss-of-function mutations in *daf-2* and *daf-16*, part of the core IIS pathway. An overexpression of *aak-2* (TOR pathway) was also included. The whole fly experiment comprised loss-of-function mutations in *InR* and *foxo*.

**Table 1:** Identifiers for the worm and fly experiments

Perturbed components (Fig. 3)	ArrayExpress ID	GEO ID	Reference (PubMed ID)	Background	Experiment	Longevity
1	E-GEOD-1762	GSE1762	15308663^68^	*daf-2* (e1370,m577)	*daf-2*(e1370,m577);*daf-16*(df-50)	Short lived
2	E-GEOD-9682	GSE9682	19079239^43^	N2 (RNAi)	*rheb-1* (RNAi)	Long lived
3				N2 (RNAi)	*let-363* (RNAi)	Long lived
4	E-GEOD-25513	GSE25513	21331044^69^	N2	*aak-2* (overexpression)	Long lived
5	E-TABM-757	NA	21694719^18^	daGAL_4>_UAS-*dInR* ^*DN*^	*dfoxo* ^*Δ/Δ*^ daGAL_4>_UAS-*dInR* ^*DN*^	Short lived

Analysis of differentially expressed genes for each experiment was performed using the R programming language. Initial quality control was performed using Relative Log Expression (RLE) values and Normalized Unscaled Standard Errors (NUSE) boxplots, part of the affyPLM package.^25^ Thus, arrays for a particular experiment were excluded from further analysis if the interquartile range (IQR) in the NUSE boxplot were outside +/–1.05 and the IQR in the RLE boxplot exceeded +/–0.2. Raw data were then normalised and summarised using the rma function,^26–28^ part of the affy package,^26,29^ followed by a quantile normalisation, part of the limma package.^30^ To identify differentially expressed genes, linear models and the empirical Bayes moderated *t*-statistic were used, as implemented in the limma package. Differentially expressed genes were identified as exhibiting an adjusted *p*-value of <0.005 for all worm experiments and <0.001 for the fly experiment (correction applied: Benjamini and Hochberg or “BH” in the limma package). Only differentially expressed genes that are part of the IIS and TOR pathways were considered in both worm and fly experiments.

### 2.3. NetEffects

In order to identify “routes” or paths that could potentially modify the longevity phenotype in the abovementioned experiments we used NetEffects as a tool for inferring the impact of differential gene expression on the IIS and TOR pathways and relating these inferences on the longevity phenotype. It has been previously developed and tested to identify paths related to longevity changes from fly mutants of the insulin/insulin-like growth factor signalling pathway.^31^ NetEffects employs Answer Set Programming (ASP), a method for declarative programming that supports logic-based inferences. NetEffects was adapted to analyse the IIS and TOR pathways in *C. elegans* in the same fashion as the equivalent pathway for the fly described in Papatheodorou *et al.* 2012.^31^ In addition, paths were classified as “primary” or “secondary” by using the perturbed or differentially expressed component/gene as starting point respectively. The web-service for the *C. elegans* version of the IIS pathway is available *via*; http://www.ebi.ac.uk/thornton-srv/software/NetEffects/worm_path.php.

## 3. Results and discussion

### 3.1. A signalling network model of the Insulin and its interaction with the TOR pathway

The single insulin receptor (*daf-2*) can be activated by at least 40 different insulin-like (*ins*) peptides,^32–34^ following signals from olfactory and chemosensory neurons. The activation of daf-2 leads to the recruitment of the insulin receptor substrate (*ist-1*),^35^ which in turn can activate the phosphoinositide-3-kinase (PI3K) complex. The PI3K complex comprises the age-1 (PI3K-like catalytic subunit) and aap-1 (PI3K-like adaptor subunit) proteins and can also be directly activated by *daf-2.* ^35^ Phospholipid products of PI3K (phosphatidylinositol(3,4,5) triphosphate – PIP3) can activate phosphoinositide-dependent kinase-1 (*pdk-1*), leading to the activation of AKT/protein kinase B-like proteins (*akt-1* and *akt-2*) and serum and glucocorticoid-inducible kinase (*sgk-1*). AKT-1, AKT-2 and SGK-1 kinases, separately and in the form a complex (protein kinase B – PKB), antagonise the activity of the forkhead transcription factor *daf-16.* ^36^

This core signal transduction pathway receives input and is modulated by a number of other proteins. These include, among others, the worm orthologue of the human *PTEN* tumour suppressor gene*, daf-18* (ref. 37) that dephosphorylates the phospholipid PIP3, thus limiting the activation of downstream AKT-1/2 kinases. AKT kinases activity is also modulated by the PP2A regulatory subunit (*pptr-1*)^38,39^ and c-Jun N-terminal kinase (JNK)-interacting protein 1 (*jip-1*).^40^ Furthermore, activated *daf-16* can inhibit the transcription of at least one of the insulin-like peptides (*ins-7*)^33^ and activate another (*ins-18*).^41^ These positive and negative feedback loops are in effect a self-regulation of the core insulin signal transduction. However, it has to be considered that transcriptional feedback operates on much slower timescale compared to signalling processes.

The insulin pathway is highly connected and interacts with several other pathways, such as ERK, p38MAPK, KGB, JNK, RAS, notch and wnt signalling. In addition, it is also well connected with the target-of-rapamycin (TOR) pathway, mainly *via* TORC2 and CeTORC1 complexes. The energy sensing AMP-activated protein kinase (*aak-2*), part of the TOR pathway, is activated by decreased energy or glucose levels (*i.e.* low AMP to ATP ratio),^42^ which in turn inhibits GTPase rheb-1, an upstream activator of CeTOR (*let-363*).^43^ The CeTORC1 and TORC2 complexes are thought to regulate fat metabolism, feeding, larval development and growth.^44,45^

### 3.2. Paths that modulate longevity in worms

We built a model of the worm IIS and TOR pathways following a literature review of the current (as of time of writing) knowledge of the different components, part of the two pathways. Connections (*i.e.* transcriptional inhibitions and activations) were compiled solely from experimental evidence, thus allowing inferences of the effect of perturbed genes to reproduce as closely as possible the paths that could be operational *in vivo*. Despite the worm pathway being as comprehensive as possible, enzymatic kinetics, mRNA levels/half lives and post-transcriptional modifications have not been included in the model. This was mainly due to a lack of comprehensive and consistent data. Moreover, correlative studies between mRNA and protein abundance suggest a relatively medium degree of correlation.^46–48^ Thus, although mRNA expression studies are somewhat easier to perform than protein identification and quantification, they are only correlative to the product of interest, *i.e.* proteins. An example of such discrepancy within the IIS pathway is a transcriptional feedback from *FOXO* to the insulin receptor *InR*, in *Drosophila* ^49^ and mammals.^50^ In these experiments transcriptionally active *FOXO* activates transcription of *InR* itself, but the two-fold increase in the *InR* mRNA does not account for the five-six fold increase in protein abundance.

Despite the basic pitfalls of this research, several important paths and transcriptional feedback loops are relatively worthy of further examination and these are described below. Although there were other paths and subpaths that are likely to have an effect on longevity, the inferred effects were considered too speculative. Therefore, such paths are described in the supplementary results (ESI†), along with graphs that illustrate the paths.

Several paths, parallel to the core insulin transduction signalling, within the worm experiments considered, were found to support or contradict the observed phenotype. If these are operational within the cells, they could enhance or diminish the longevity effect of core insulin signalling *via daf-16*. At least according to our model, these paths converge on two transcription factors, *i.e. hif-1* and *skn-1*.

#### 3.2.1 Longevity path *via hif-1*

Lifespan extension of stabilised hypoxia-induced transcription factor *hif-1* appears to be IIS independent^51^ and deletions of *hif-1* have an effect on longevity in a temperature-dependent manner^52^ and are *daf-16* dependent. Four of the experiments (*daf-2 vs. daf-2*;*daf-16*, N2 *vs. aak-2* oe, N2 *vs. let-363*i and N2 *vs. rheb-1*i) exhibited a common subpath in their primary effect (path starts from the perturbed component/gene). This path comprised an activation of the ribosomal protein S6 kinase (*rsks-1*) by CeTORC1 complex, which in turn activates the hypoxia-induced factor *(hif-1*) and a potential *hif-1* mediated increase in longevity (Fig. S1, S6 and S14, ESI†). The signalling in this particular common path in some of the experiments was likely increased (*daf-2 vs. daf-2*;*daf-16*) and in others decreased (N2 *vs. aak-2 oe*, N2 *vs. let-363*i and N2 *vs. rheb-1*i), but in all of the four experiments this path contradicted the observed phenotype.

Thus in the long-lived experiments (*rheb-1*i and *let-363*i) a decreased activity of the CeTORC1 complex is likely to lead to a decreased activity of *hif-1* and a potential *hif-1*-mediated decrease in longevity. It has to be said that although an inhibition from *hif-1* to *daf-16* is recorded in the worm pathway model, the current version of NetEffects only displays the shortest path from the perturbed components to longevity. While not displayed in NetEffects, the likely outcome from a reduced *hif-1* activity would be a decreased inhibition of *daf-16* and possible *daf-16*-mediated lifespan extension, as previously shown under normoxic conditions.^52^ In the short-lived *daf-2*;*daf-16* experiment, a path comprising an activated CeTORC1 complex and a stabilised *hif-1* (Fig. S1, ESI†), following a knock-out of *daf-16*, is likely to oppose the observed reduction in longevity. It would be interesting to investigate if removing such a path (*e.g. hif-1* RNAi), would result in further reduction in lifespan. Additionally, for this particular experiment a likely *skn-1* mediated increase in longevity was also observed, due to the down-regulation of *akt-1/2* kinases, although a compensatory *gsk-3* inhibition of *skn-1* was also likely to be increased (Fig. S4, ESI†).

#### 3.2.2. Longevity paths *via skn-1*

The transcription factor *skn-1* is a stress-response gene that activates Phase 2 detoxification response^53^ and mainly exerts its effect in the intestine.^54^ Several lines of evidence suggest that the effect of activated *skn-1* could potentially modulate ageing and this could be achieved in *daf-16* independent manner. Our results suggest that a primary path that leads to an increased longevity for two of the experiments (N2 *vs. let-363*i and N2 *vs. rheb-1*i) involves *skn-1*. This path exhibits a decreased activity of the CeTORC1 complex, subsequent decrease in the inhibitory link to *skn-1* and *daf-16* and an increase in longevity, mediated by *daf-16* and/or *skn-1* (Fig. S5 and S9, ESI†). It is not obvious if the increased longevity in both experiments is due to the synergistic nucleic accumulation of both transcription factors. The *sek-1* kinase, part of the p38 mitogen-activated protein kinase (MAPK), has been shown to be required for *skn-1* activation.^11^ Thus, a lifespan analysis of double mutants (*rheb-1*i;*sek-1*i and *let-363*i;*sek-1* as compared to single mutants of *rheb-1*i and *let-363*i) is likely to shed some light on the relative importance of *skn-1* and *daf-16* with respect to the effect on longevity from decreased activity of the TOR pathway. Furthermore, two kinases *sek-1* and *jnk-1*, part of the p38 and JNK MAPKs, were up-regulated in both experiments, suggesting a transcriptional feedback from TOR pathway to JNK. These up-regulated kinases led to several paths that were likely responsible for the *skn-1* and *daf-16* mediated increase in longevity, although this does not preclude the possibility of other TOR-mediated independent of *skn-1* and *daf-16* longevity mechanisms, such as a reduction of mRNA translation, increased autophagy or *via* the germline.^55–57^ An experiment of inhibited activity of *sek-1* kinase would also likely reduce the activation of *skn-1* by *pmk-1* ^58^ and the activation of *daf-16* by *jnk-1.* ^59^ If such experiments are performed, according to our worm IIS and TOR pathways model, the increased longevity phenotype of *rheb-1*i and *let-363*i, would only result from a decreased inhibition of *daf-16* by the TOR pathway and a survival analysis would point the relative contribution of *skn-1*. In support of such hypothesis comes a study by Tullet *et al.* 2008 that has suggested an independent of *daf-16* pro-longevity phenotype of *skn-1*. Although, as both transcription factors compete for binding to the negative regulators *akt-1*, *akt-2* and *sgk-1*, elimination of either one could result in availability of these negative regulators.^11^

In these two experiments (*rheb-1*i and *let-363*i), several paths that contradicted the increase in longevity were also revealed (Fig. S8 and S12, ESI†). Some were due to a likely increased IIS signalling by up-regulated insulin-like peptides. As the observed phenotype was an increase in longevity, these paths were found to contradict this phenotype. It would be interesting to investigate the relative roles of increased IIS signalling and decreased TOR signalling, with respect to the increase in longevity. Similarly to the previous experiments proposed, a double (*daf-2*;*rheb-1* and *daf-2*;*let-363*) mutant could help delineate the relative contribution of the increased IIS signalling and reduced TOR signalling, with respect to the longevity phenotype.

### 3.3. A comparison with fly IIS and TOR signal transduction pathways (*InR vs. InR*;*foxo*)

A direct comparison between the IIS and TOR pathways in both flies and worms reveals that the two pathways are in general evolutionarily conserved. The core components of the IIS are present in both organisms, including the insulin/IGF-like receptor, PI3K, PDK, AKT signal transduction kinases and the forkhead box O (foxo) transcription factor. Differences are also noticeable. There are at least 40 different insulin-like peptides in the worm as compared to only eight in the fly. These insulin-like peptides agonise the sole insulin/IGF receptor (*InR*) in the fly, where at least one insulin-like peptide (*ins-18*) in worms has been shown to antagonise *daf-2* ^41^ and transcriptional feedback loops from *daf-16* to *daf-2* have been shown. The majority of components part of the TOR pathway are also relatively conserved across the worm and fly. The TORC1/2 complexes are both present, although there is not a worm orthologue of the tuberous sclerosis complex (TSC).

As previously mentioned, the IIS and TOR pathways are evolutionarily conserved in fly and worm.^6,19^ However, having experiments of orthologous components in both the fly and worm, now allowed us to compare the differences in the two pathways and the effect on longevity.

**Fig. 2:**
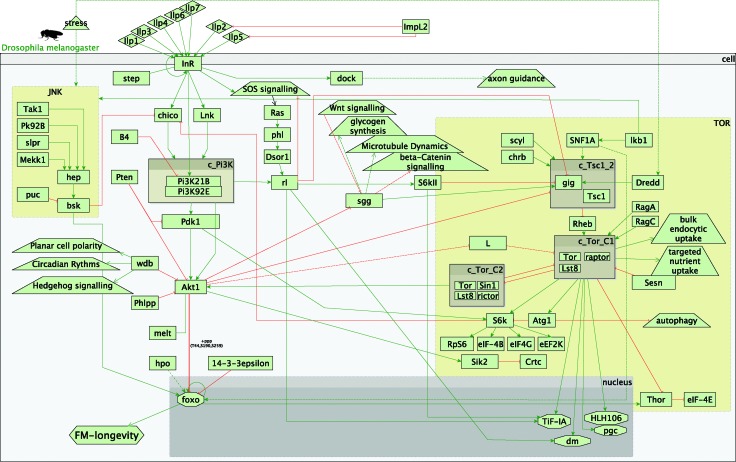
Overview of the fly insulin and TOR pathways. Legend: rectangles represent genes; diamonds – molecules; triangles – environmental effects; trapezoids – other than IIS or TOR pathways; octagons – transcription factors; green arrow lines represent activation; red t-shaped lines represent inhibition; brown boxes starting with c_ represent complexes.

In the fly experiment (*InR vs. InR*;*foxo*) all possible paths (*i.e.* primary and secondary) led only to a decrease in longevity, supporting the observed phenotype (Fig. S17 and S18, ESI†). This was to be expected, as in the IIS/TOR model in flies, longevity is recorded as only modulated *via* foxo and foxo is down-regulated in the experiment (Fig. 2).

**Fig. 3:**
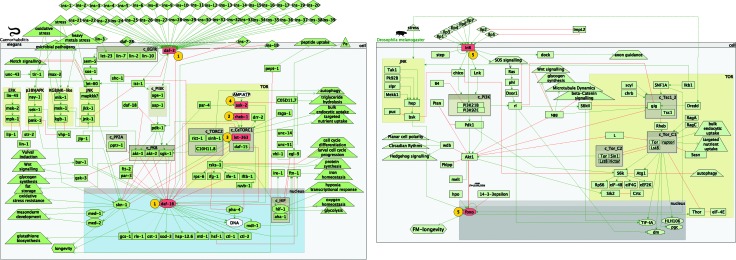
Perturbed components in the worm and fly experiments.

On the other hand, in our worm signalling model there are several possible inputs into longevity. These comprise *daf-16* (orthologue of the fly *foxo*), *skn-1* and *hif-1*. Thus, other paths that decrease longevity in the worm were observed, apart from the *daf-16*/*foxo* mediated decrease in longevity, which was observed in both experiments.

Several paths from the TOR pathway and the interaction with IIS were observed to complement the observed decrease in longevity (Fig. S17 and S18, ESI†). These paths were mainly down-regulating *S6k*, followed by a decreased inhibition of *chico* and increased IIS signalling. While an interaction between the IIS and TOR pathways was shown in the worm experiment (reduced inhibition of CeTORC1 complex by *daf-16*, Fig. S1, S3 and S4, ESI†), this was not *via rsks-1*, the worm orthologue of *S6k*. Furthermore, in the worm, the transcription factors *skn-1* and *hif-1* were part of paths that were likely to lead to an increase in longevity as compared to paths that would result only in decrease in longevity in the fly experiment.

### 3.4. Transcriptional feedback in insulin signalling

The primary target of the worm and fly insulin-like peptides is the sole insulin receptor^34^ *InR* in flies and *daf-2* in worms,^34^ part of the core IIS signal transduction pathway to ultimately inhibit the nuclear translocation of the transcription factor *daf-16/foxo*.

For several of the microarray experiments, a consistent transcriptional feedback to the insulin-like peptides was evident. In the long-lived experiments (*rheb-1*i and *let-363*i), there was a substantial number of up-regulated insulin-like peptides (seven and nine in the *let-363* and *rheb-1* respectively), as compared to the wild-type (N2). In these two worm experiments out of the 40 there were altogether nine up-regulated insulin-like peptides and seven of those were found in both experiments. The overall effect of the up-regulated insulin-like peptides would be an increase in the core insulin signal transduction, thereby leading to an overall inhibition of *daf-16* and/or *skn-1*, followed by a potential *daf-16* and/or *skn-1* mediated decrease in longevity.

Several insulin-like peptides were down-regulated in the *daf-2*;*daf-16* double mutant experiment as compared to *daf-2* background (*ins-33* and *ins-35*). Hence, the down-regulated insulin-like peptides in this short-lived experiment would ultimately reduce the core insulin signalling transduction. However, since *daf-2* and *daf-16* are inhibited in this experiment, the transcriptional feedback would appear dysfunctional.

The down-regulated insulin-like peptides could potentially be explained by a positive feedback loop from *daf-16*. This has been shown for *ins-18*,^41^ but crucially not for any of the other up-regulated peptides pertinent to the results obtained in this work.

In addition, the two of the perturbed components (*i.e. rheb-1* and *let-363*) are part of the TOR pathway. Therefore, if a transcriptional feedback from *daf-16* to the insulin-like peptides is indeed active then the effect of the perturbed components (*i.e. rheb-1* and *let-363*) must converge on *daf-16*. Indeed, several paths within these two experiments show a decreased inhibition of *daf-16* by the CeTORC1 complex. Thus, a transcriptionally active *daf-16* could provide a positive feedback loop to the insulin-like peptides.

Conversely, a negative transcriptional feedback in the *daf-2*;*daf-16* double mutant experiment that contradicted the observed phenotype, was also observed. The *ins-7* was up-regulated and the likely outcome would be an overall increase in the core insulin signalling and a likely *daf-16* and/or *skn-1* mediated decrease in longevity. A previously suggested negative transcriptional feedback from *daf-16*, could explain the observed up-regulation of *ins-7.* ^33^

In the fly experiment two of the Ilps were also down-regulated (*Ilp3* and *Ilp6*), nonetheless there is a paucity of experimental evidence to suggest a transcriptional feedback from the IIS similar to the worm. Still, it has been suggested that a positive feedback loop from fly *foxo* could result in the up-regulation of *Ilp3* and *Ilp5* ^60^ and *Ilp3* was found to be up-regulated in the fly *InR*;*foxo* experiment.

Despite several lines of evidence, suggesting a transcriptional feedback loop from *daf-16*/*foxo*, it is also possible that a signal other than *daf-16* or *foxo* could provide a transcriptional feedback loop to the insulin-like peptides. Several of the paths examined intersect on the transcription factor *skn-1*, shown to inhibit one of insulin-like peptides (*ins-7*)^33,61^ and *skn-1* suppression coupled with a *daf-28* induction has been suggested as a negative transcriptional feedback.^62^ Furthermore, both *ins-7* and *daf-28* were found to be up-regulated in 2 of the experiments, *i.e. rheb-1*i and *let-363*i. As a result, it is possible that differential expression of at least some of the insulin-like peptides in these experiments is due to *skn-1*.

As previously argued, the effects of the inhibition of *rheb-1* and *let-363* RNAi experiments, could converge on *daf-16* and/or *skn-1*, hence providing positive or negative feedback to the insulin-like peptides. This could explain the up- and down-regulated insulin-like peptides in the double mutant *daf-2*;*daf-16*. However, *ins-7*, *daf-28* and several other insulin-like peptides were found to be down-regulated in the *rheb-1* and *let-363* experiments. By this means if, *ins-7* and *daf-28* are indeed under negative transcriptional control of *daf-16*/*skn-1* and the others under positive control, both up and down-regulated insulin-like peptides would be expected, similarly to the double mutant *daf-2*;*daf-16*.

Therefore, other mechanisms could also be responsible for the differential insulin-like peptides regulation, similar to a gene dosage effect of *ins-18* over other insulin-like genes.^34^

## 4. Conclusions

*Drosophila melanogaster* and *Caenorhabditis elegans* diverged ∼990 million years ago.^63^ Thus, it is not surprising that several cellular mechanisms and physiological processes exhibit substantial differences between flies and worms. Flies have relatively well differentiated brain, a heart, vascular system, vision and hearing, where worms do not. In terms of cellular processes, phagocytosis in *C. elegans* for example, does not seem to play a role in microorganism clearance. On the other hand apoptotic pathway defence response in flies has not yet been reported within the innate immune response.^64^ The sensory system in flies is substantially more complex than in worms. It has been proposed that there are at least two sensory-lifespan pathways present in flies with its genes expressed in different sensory neurons, in contrast to worms (for a detailed review see Linford *et al.* 2011^65^).

A general overview of the IIS and TOR pathways in the worm and fly suggests remarkable similarities. This signifies the importance of the nutrient sensing properties of the IIS and TOR pathways. Even though both species occupy distinct ecological niches, the molecular basis of nutrient sensing is similar over large evolutionary distance. The core IIS signal transduction pathway appears evolutionary conserved, although some differences are noticeable. A major difference is the absence of the *Drosophila* insulin receptor substrates (IRS) *chico* and *Lnk* and the absence of the TSC complex, part of the TOR pathway, although an equivalent IRS homolog, *ist-1*, is present in worms. Unfortunately, due to the absence of corresponding experiments in the fly we could not show similarities and/or differences in the paths leading to longevity, apart from the *foxo*-mediated effect on longevity. There were some indications of conserved transcriptional feedback mechanisms that are very similar in both model organisms. That is, a transcriptional feedback from *foxo* to the insulin-like peptides. One major difference that would greatly influence any such comparison is the lack of different factors recorded in our fly pathway model that could mediate longevity, apart from *foxo*. It is possible that some of these factors are worm-specific. The hypoxia-induced factor *hif-1* is evolutionary conserved and present in worms, flies and mammals,^66^ thus it is very likely performing a homologous function similar to worms, although a *hif* mediated effect on longevity in flies remains to be seen. On the other hand, the stress response gene *skn-1* in worms appears to be without a homolog in *Drosophila*, thus it is likely to be worm-specific, although functional counterparts in mammals have been previously described.^67^

The work presented here indicates that such approach is useful in analysing the effects of perturbed components within the IIS and TOR pathways, with respect to the effect on longevity. Furthermore, this approach could be extended, as new knowledge is accumulated, to other pathways that have an effect on lifespan or biological processes, such as autophagy.
