# Supplementary material for: Transcriptional feedback in the insulin signalling pathway modulates ageing in both Caenorhabditis elegans and Drosophila melanogaster
Source: Mol Biosyst. 2013 Apr 26;9(7):1756–64. doi: 10.1039/c3mb25485b (PMC3693544; doi:10.1039/c3mb25485b)
Supplement: Supplementary file 1 [file MB-009-c3mb25485b-s001.pdf]

This journal is © The Royal Society of Chemistry 2013

1. Supplementary material and methods

1.1. NetEffects

The web-service for the *C. elegans* version of the IIS pathway is available via http://www.ebi.ac.uk/thornton-srv/software/NetEffects/worm_path.php. It provides the user with two options. One option involves performing queries on the pathway and exploring the impact on longevity. For example, the user can theoretically overexpress and/or knock out a gene or a set of genes of selected pathway components to observe the inferred alterations in the pathway and how these may impact longevity. The second option involves the upload of a gene expression data set, as well as background information on the experiment, such as details on gene mutation and phenotype. NetEffects will then show which genes from the IIS and TOR pathways are differentially expressed and whether these are up- or down-regulated. It will then infer any downstream paths for each one of these genes to the node "longevity", thereby showing the potential influence of each one of these experimental effects of differential gene expression to the longevity phenotype.

2. Supplementary Results

Several paths that are not included in main text, but could modulate longevity, are described below.

2.1. Core insulin signal transduction path

In all of the experiments paths that are part of the core insulin signal transduction were detected. For some of the experiments, this was a primary effect (*daf-2* vs. *daf-2;daf-16*) and for others a secondary effect (N2 vs. *aak-2* oe and N2i vs. *rheb-1*i N2 vs. *let-363*i). For the *daf-2* vs. *daf-2;daf-16*experiment, the primary effect from a down-regulated *daf-2* contradicted the observed phenotype via a decreased inhibition of *skn-1* by *akt-1/2* (Supplementary Figure 1).
The secondary effect in the two experiments in *rheb-1* and *let-363* from up-regulated insulin-like peptides to a decreased expression level of *daf-16* contradicted the observed phenotype of increased longevity (Supplementary Figures 8 and 12).

In contrast, a decreased expression level of the *ins-35* in N2 vs. *aak-2* (oe) was observed, leading to a likely up-regulation of *daf-16* and potential *daf-16* mediated increase in longevity, which supported the observed phenotype (Supplementary Figure 15).

2.2. Other paths

2.2.1. Signal transduction path via *let-60* and *skn-1*

For all of the experiments a primary or secondary path was observed, starting from *daf-2* or the insulin-like peptides activating *ist-1* and *let-60* and subsequently a potential *skn-1* mediated modulation of longevity via activation by *let-60* (Supplementary Figure 2, 4, 7, 8, 11, 12 and 16). This path appears to be functional in parallel to the insulin core signal transduction. This path contradicted the observed phenotype in two of the experiments (*daf-2* vs. *daf-2;daf-16* and N2 vs. *aak-2* oe; Supplementary Figures 2, 4 and 16) and supported the observed phenotype in both experiments in N2 vs. *let-363*i and N2 vs. *rheb-1*i (Supplementary Figures 7, 8, 11 and 12).

2.2.2. Signal transduction path via *pmk-1*

An increase or reduction in the activation of *skn-1* and a potential *skn-1* mediated increase in longevity by *pmk-1* was observed in two of the experiments (N2 vs. *rheb-1*i and N2 vs. *let-363i*). The path starts from an activation of *pmk-1* by up-regulated *sek-1* (Supplementary Figures 7 and 11). The path in both experiments supported the observed phenotype, leading to an increase in *skn-1* mediated longevity.

2.2.3. Signal transduction path via *jnk-1*

In two of the experiments *jnk-1*, part of the c-Jun N-terminal kinase complex (JNK), was up-regulated by the up-regulated *sek-1* component of the p38 mitogen-activated protein kinase complex (p38MAPK) and is likely to lead to up-regulation of *daf-16* and *daf-16* mediated increase in longevity. These experiments were N2 vs. *rheb-1*i and N2 vs. *let-363*i (Supplementary Figures 7 and 11). This path was a secondary path, i.e. starting from a

This journal is © The Royal Society of Chemistry 2013

differentially expressed component, and for both of the experiments supported the observed increase in longevity.

2.2.4. Signal transduction path via *sgk-3*

In three of the experiments, a secondary path from the protein kinase-B complex (PKB) via *gsk-3* is likely to lead to *skn-1* mediated modulation of longevity. The experiments included GSE9682 (N2i vs. *rheb-1*i and N2i vs. *let-363*i) and GSE1762 (*daf-2* vs. *daf-2;daf-16*). For all of these experiments the path supported the observed phenotype (Supplementary Figures 4, 7 and 11).

This journal is © The Royal Society of Chemistry 2013

3. Supplementary Figures

All signal transduction paths that were generated in this research are given below.

Legend: Rectangles represent genes; Diamonds- molecules; Triangles- environmental effects; Trapezoids- other than IIS or TOR pathways; Octagons- transcription factors; Green arrow lines represent activation; Red t-shaped lines represent inhibition; Brown boxes starting with c_ represent complexes;

Electronic Supplementary Material (ESI) for Molecular BioSystems
This journal is © The Royal Society of Chemistry 2013

Supplementary Figure 1 Primary effect (increase in longevity), *daf-2* vs. *daf-2;daf-16*

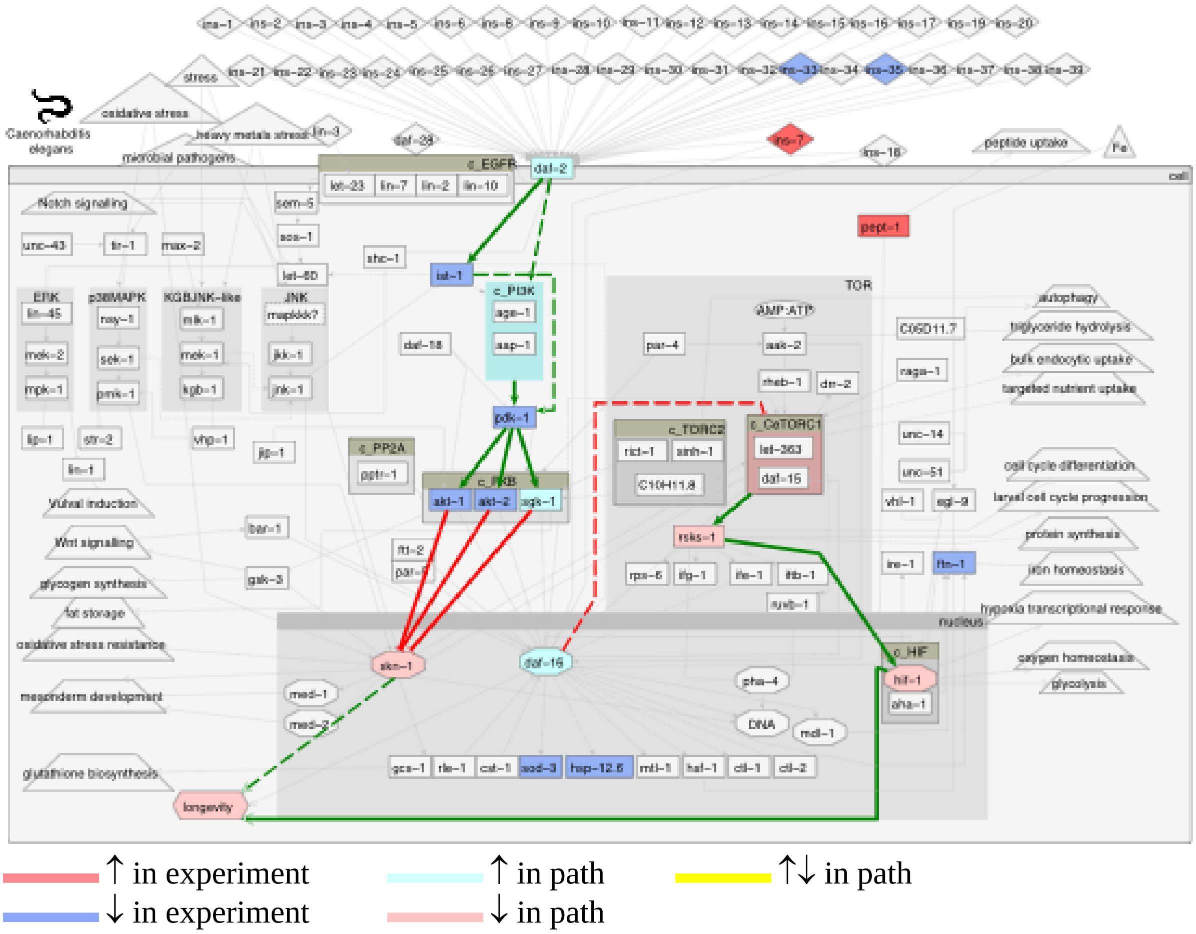

Primary effects ↑Longevity					
daf-16 ↓	c_CeTORC1 ↑	rsks-1 ↑	hif-1 ↑	longevity ↑	
daf-2 ↓	c_PI3K ↓	pdk-1 ↓	akt-1 ↓	skn-1 ↑	longevity ↑
daf-2 ↓	c_PI3K ↓	pdk-1 ↓	akt-2 ↓	skn-1 ↑	longevity ↑
daf-2 ↓	c_PI3K ↓	pdk-1 ↓	sgk-1 ↓	skn-1 ↑	longevity ↑
daf-2 ↓	ist-1 ↓	pdk-1 ↓	akt-1 ↓	skn-1 ↑	longevity ↑
daf-2 ↓	ist-1 ↓	pdk-1 ↓	sgk-1 ↓	skn-1 ↑	longevity ↑
daf-2 ↓	ist-1 ↓	pdk-1 ↓	akt-2 ↓	skn-1 ↑	longevity ↑

Electronic Supplementary Material (ESI) for Molecular BioSystems
This journal is © The Royal Society of Chemistry 2013

Supplementary Figure 2 Primary effect (decrease in longevity), *daf-2* vs. *daf-2;daf-16*

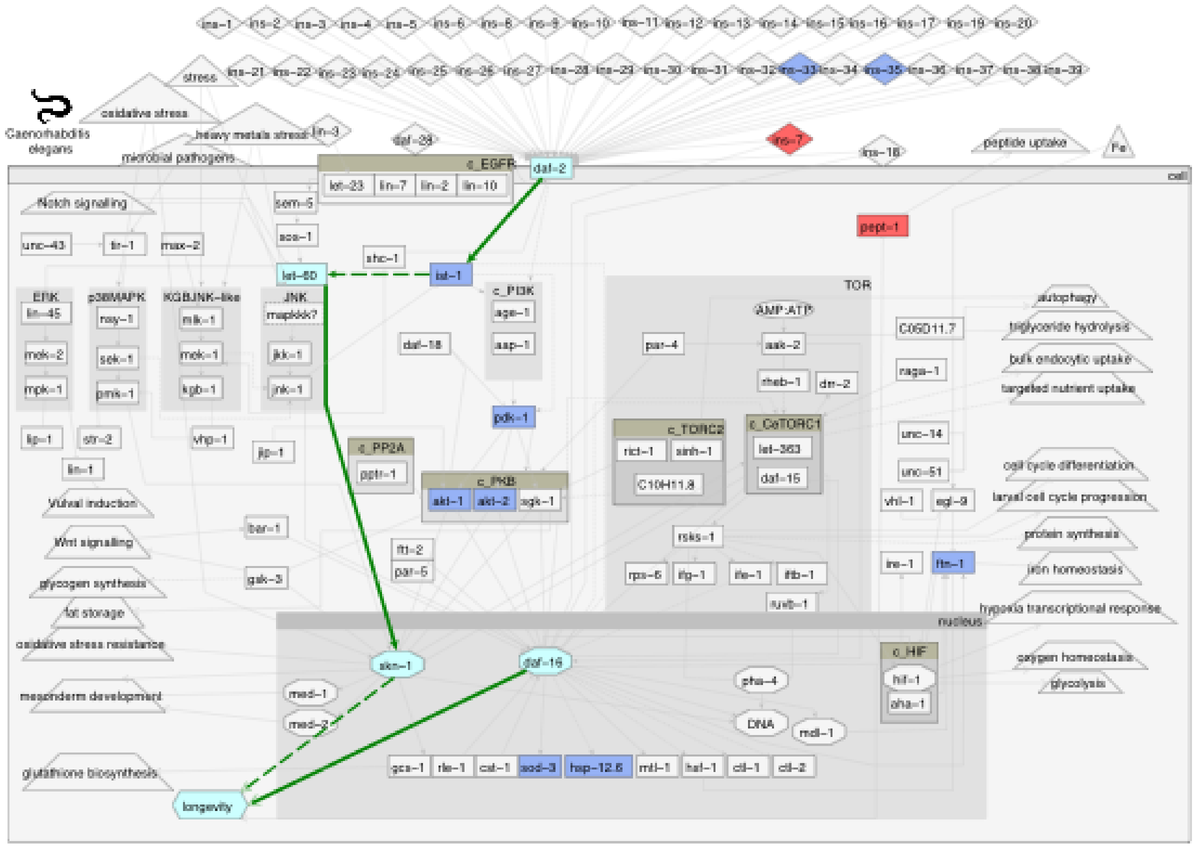

↑ in experiment
↓ in experiment
↑ in path
↓ in path
↑↓ in path

Primary effects ↓Longevity						
daf-16 ↓	longevity ↓					
daf-2 ↓	ist-1 ↓	let-60 ↓	skn-1 ↓	longevity ↓		

Supplementary Figure 3 Secondary effect (increase in longevity), *daf-2* vs. *daf-2;daf-16*

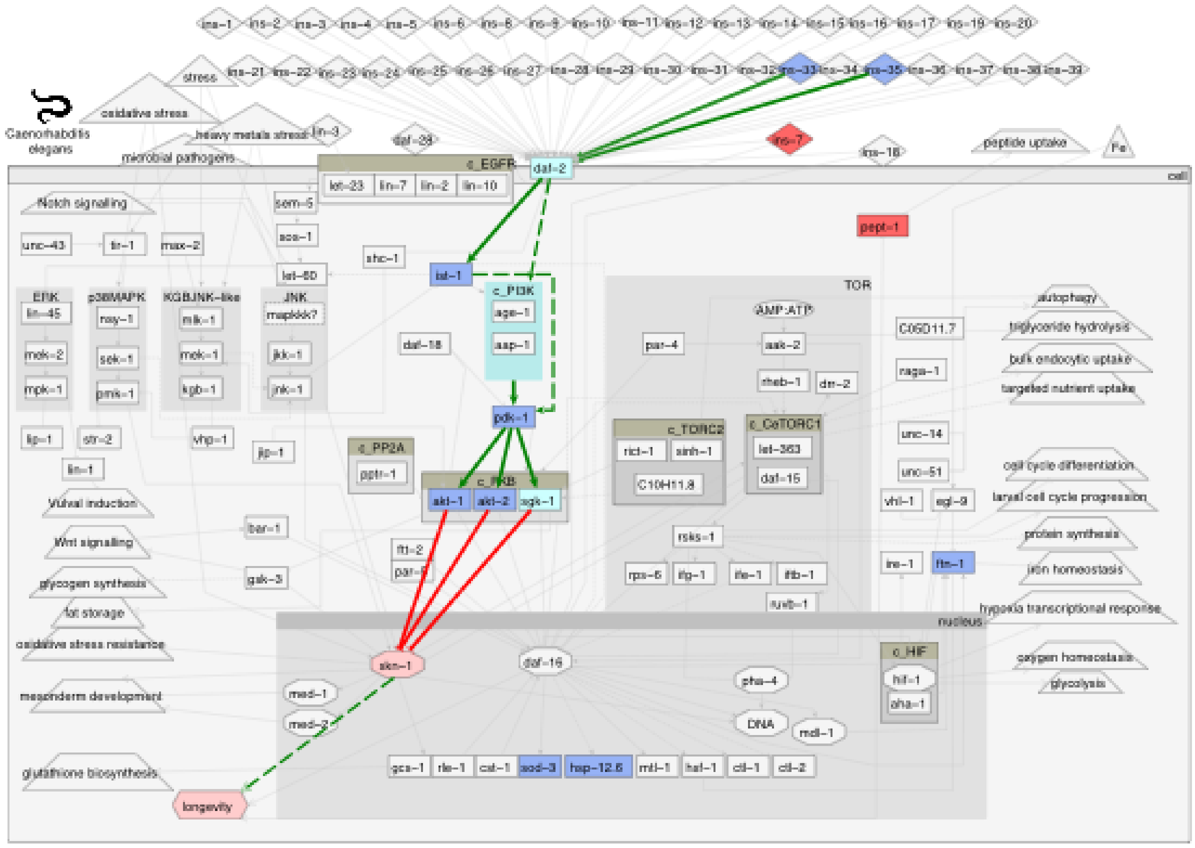

↑ in experiment	↑ in path
↓ in experiment	↓ in path
↑↓ in path	

Secondary effects ↑Longevity						
akt-1 ↓	skn-1 ↑	longevity ↑				
akt-2 ↓	skn-1 ↑	longevity ↑				
pdk-1 ↓	sgk-1 ↓	skn-1 ↑	longevity ↑			
pdk-1 ↓	akt-1 ↓	skn-1 ↑	longevity ↑			
pdk-1 ↓	akt-2 ↓	skn-1 ↑	longevity ↑			
ist-1 ↓	pdk-1 ↓	akt-2 ↓	skn-1 ↑	longevity ↑		
ist-1 ↓	pdk-1 ↓	akt-1 ↓	skn-1 ↑	longevity ↑		
ist-1 ↓	pdk-1 ↓	sgk-1 ↓	skn-1 ↑	longevity ↑		
ins-35 ↓	daf-2 ↓	c_PI3K ↓	pdk-1 ↓	akt-1 ↓	skn-1 ↑	longevity ↑
ins-35 ↓	daf-2 ↓	c_PI3K ↓	pdk-1 ↓	akt-2 ↓	skn-1 ↑	longevity ↑
ins-35 ↓	daf-2 ↓	c_PI3K ↓	pdk-1 ↓	sgk-1 ↓	skn-1 ↑	longevity ↑
ins-33 ↓	daf-2 ↓	c_PI3K ↓	pdk-1 ↓	akt-1 ↓	skn-1 ↑	longevity ↑
ins-33 ↓	daf-2 ↓	c_PI3K ↓	pdk-1 ↓	akt-2 ↓	skn-1 ↑	longevity ↑
ins-33 ↓	daf-2 ↓	c_PI3K ↓	pdk-1 ↓	sgk-1 ↓	skn-1 ↑	longevity ↑
ins-35 ↓	daf-2 ↓	ist-1 ↓	pdk-1 ↓	akt-1 ↓	skn-1 ↑	longevity ↑
ins-35 ↓	daf-2 ↓	ist-1 ↓	pdk-1 ↓	akt-2 ↓	skn-1 ↑	longevity ↑
ins-35 ↓	daf-2 ↓	ist-1 ↓	pdk-1 ↓	sgk-1 ↓	skn-1 ↑	longevity ↑
ins-33 ↓	daf-2 ↓	ist-1 ↓	pdk-1 ↓	akt-1 ↓	skn-1 ↑	longevity ↑
ins-33↓	daf-2 ↓	ist-1 ↓	pdk-1 ↓	akt-2 ↓	skn-1 ↑	longevity ↑
ins-33 ↓	daf-2 ↓	ist-1 ↓	pdk-1 ↓	sgk-1 ↓	skn-1 ↑	longevity ↑

Electronic Supplementary Material (ESI) for Molecular BioSystems
This journal is © The Royal Society of Chemistry 2013

Supplementary Figure 4 Secondary effect (decrease in longevity), *daf-2* vs. *daf-2;daf-16*

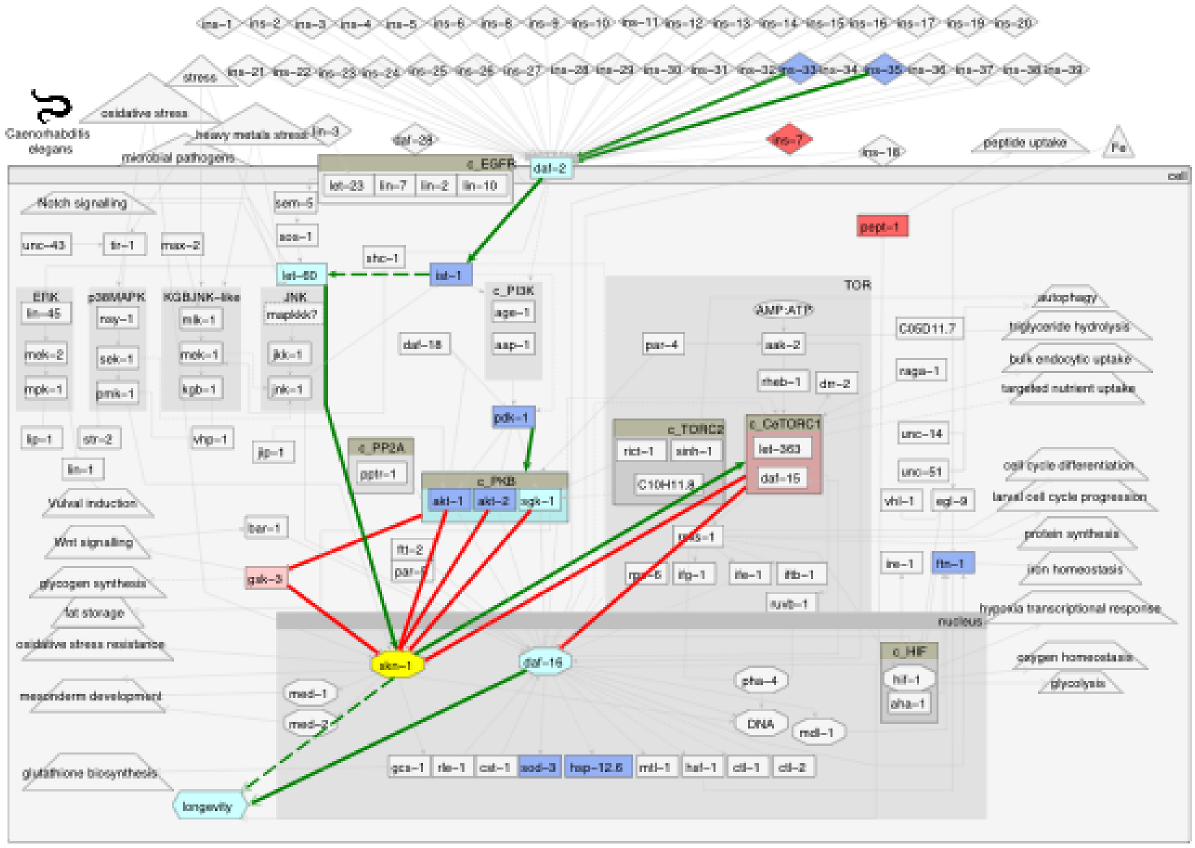

↑ in experiment
↓ in experiment
↑ in path
↓ in path
↑↓ in path

Secondary effects ↓Longevity						
ist-1 ↓	let-60 ↓	skn-1 ↓	longevity ↓			
akt-1 ↓	skn-1 ↑	c_CeTORC1 ↑	daf-16 ↓	longevity ↓		
akt-1 ↓	skn-1 ↑	c_CeTORC1 ↑	skn-1 ↓	longevity ↓		
akt-2 ↓	skn-1 ↑	c_CeTORC1 ↑	daf-16 ↓	longevity ↓		
akt-2 ↓	skn-1 ↑	c_CeTORC1 ↑	skn-1 ↓	longevity ↓		
sgk-1 ↓	skn-1 ↑	c_CeTORC1 ↑	daf-16 ↓	longevity ↓		
sgk-1 ↓	skn-1 ↑	c_CeTORC1 ↑	skn-1 ↓	longevity ↓		
akt-1 ↓	c_PKB ↓	gsk-3 ↑	skn-1 ↓	longevity ↓		
pdk-1 ↓	c_PKB ↓	gsk-3 ↑	skn-1 ↓	longevity ↓		
akt-2 ↓	c_PKB ↓	gsk-3 ↑	skn-1 ↓	longevity ↓		
sgk-1 ↓	c_PKB ↓	gsk-3 ↑	skn-1 ↓	longevity ↓		
ins-33 ↓	daf-2 ↓	ist-1 ↓	let-60 ↓	skn-1 ↓	longevity ↓	
ins-35 ↓	daf-2 ↓	ist-1 ↓	let-60 ↓	skn-1 ↓	longevity ↓	

Supplementary Figure 5 Primary effect (increase in longevity), N2i vs. *rheb-1*i

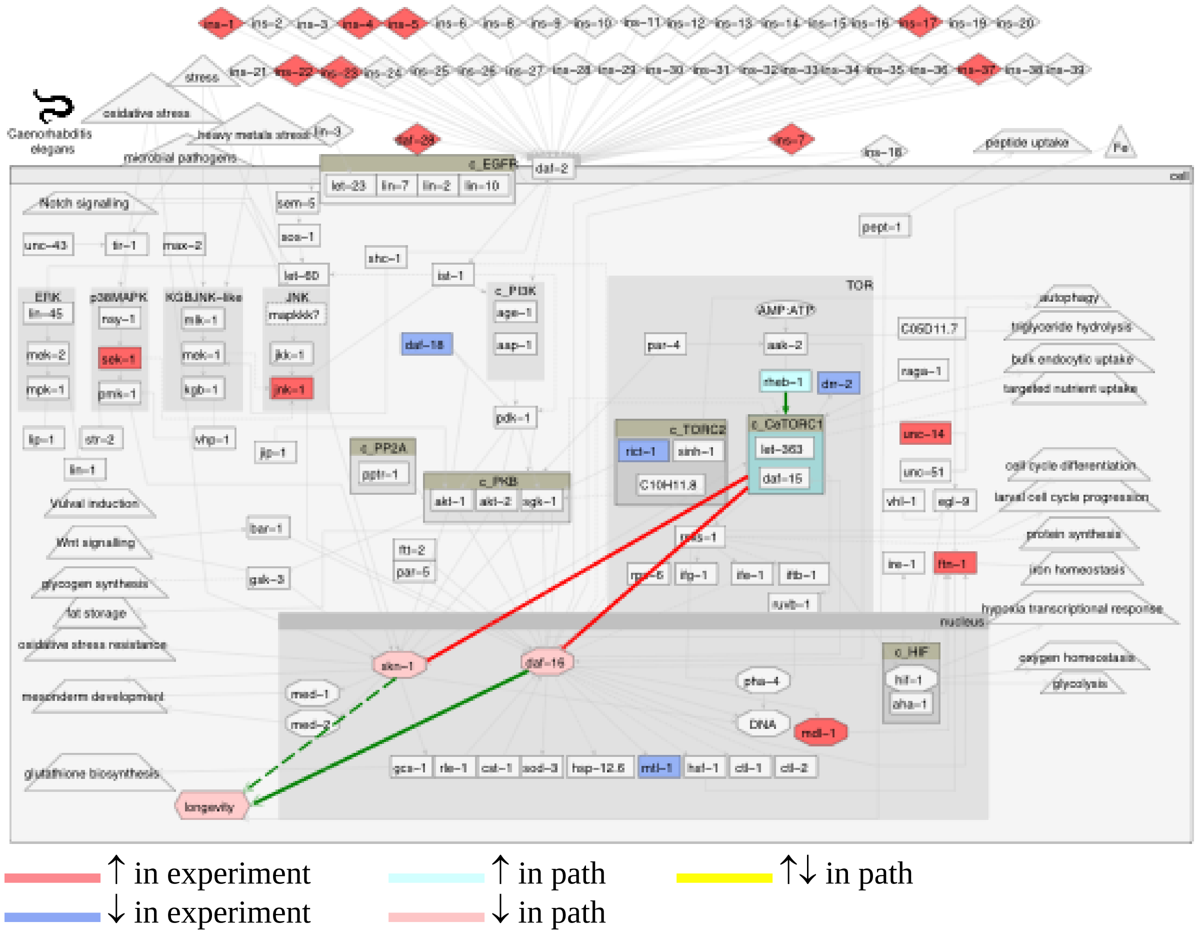

Primary effects ↑Longevity						
rheb-1 ↓	c_CeTORC1 ↓	daf-16 ↑	longevity ↑			
rheb-1 ↓	c_CeTORC1 ↓	skn-1 ↑	longevity ↑			

Supplementary Figure 6 Primary effect (decrease in longevity), N2i vs. *rheb-1*i

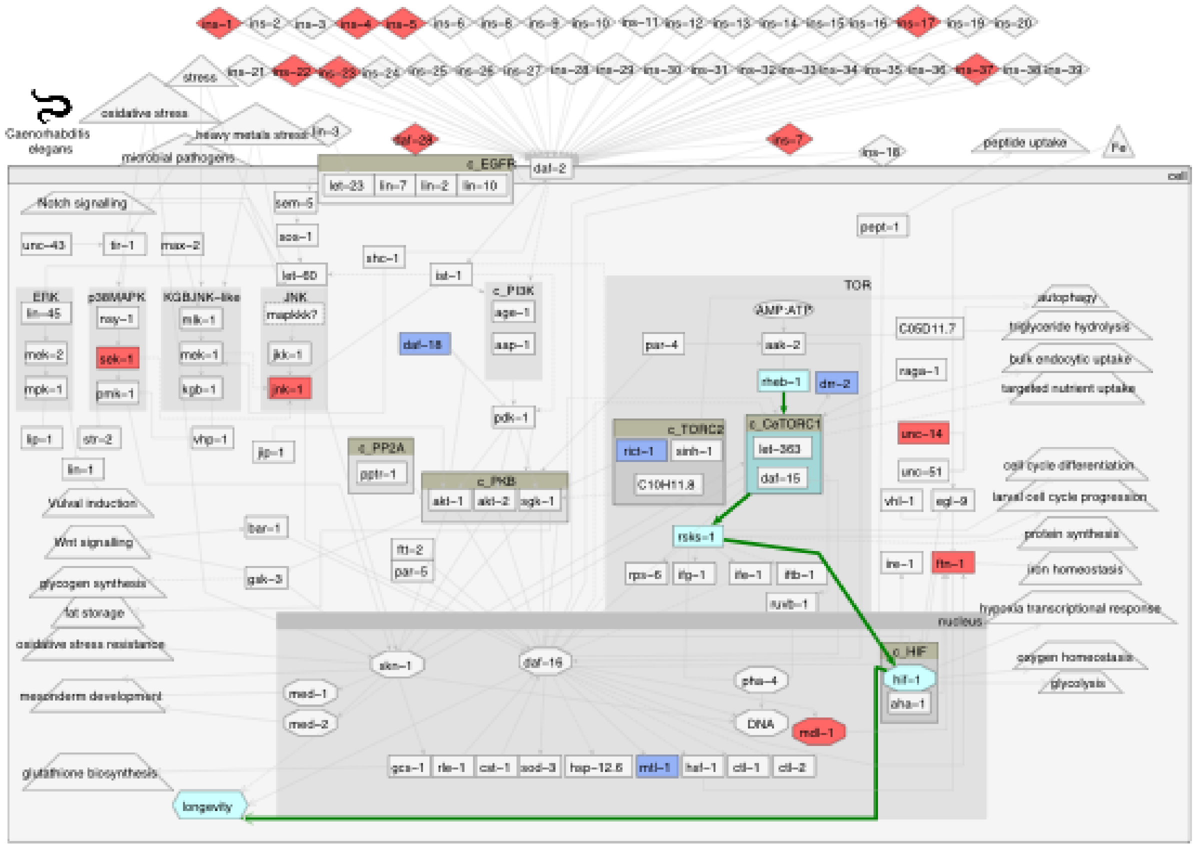

↑ in experiment
↓ in experiment
↑ in path
↓ in path
↑↓ in path

Primary effects ↓Longevity						
rheb-1 ↓	c_CeTORC1 ↓	rsks-1 ↓	hif-1 ↓	longevity ↓		

Electronic Supplementary Material (ESI) for Molecular BioSystems
This journal is © The Royal Society of Chemistry 2013

Supplementary Figure 7 Secondary effect (increase in longevity), N2i vs. *rheb-1*i

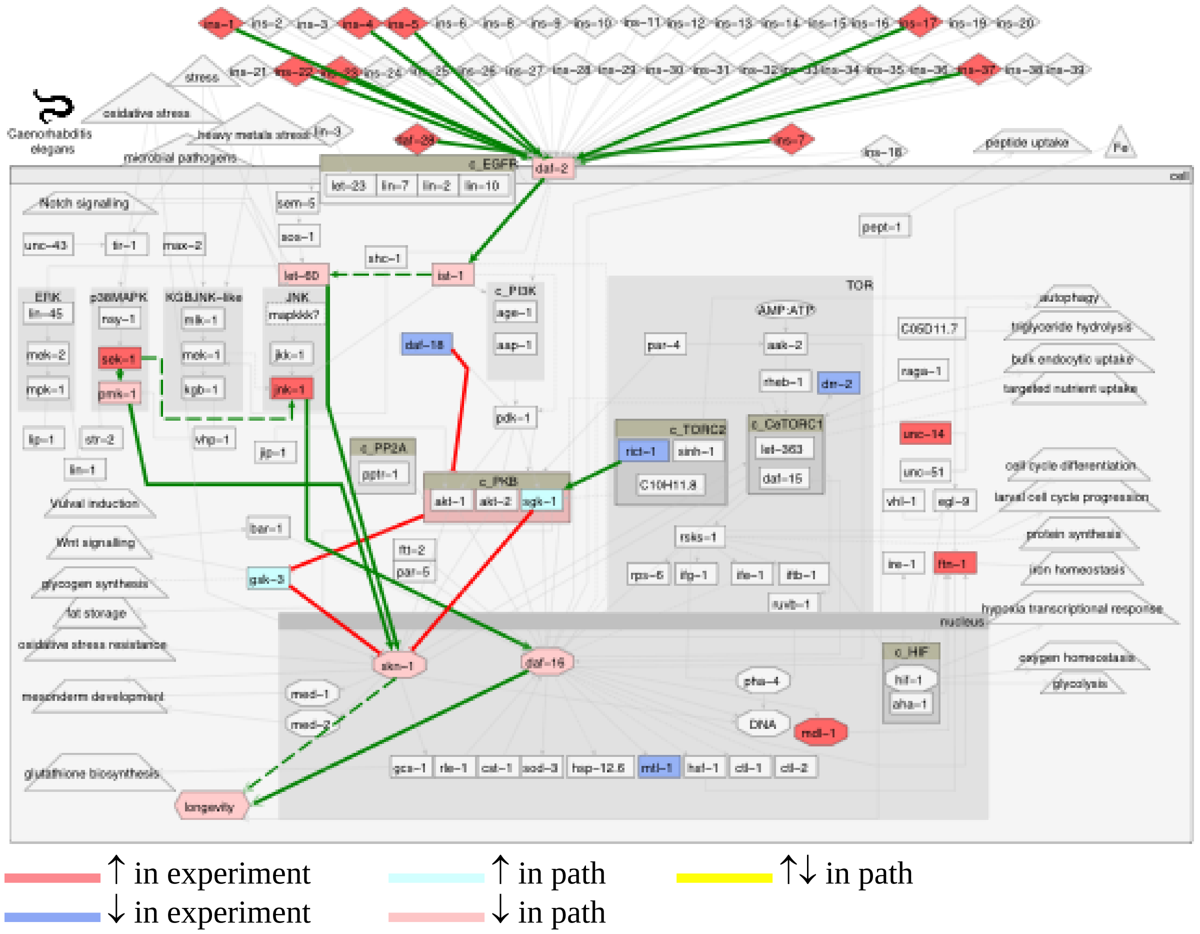

Secondary effects ↑Longevity						
jnk-1 ↑	daf-16 ↑	longevity ↑				
sek-1 ↑	jnk-1 ↑	daf-16 ↑	longevity ↑			
sek-1 ↑	pmk-1 ↑	skn-1 ↑	longevity ↑			
rict-1 ↓	sgk-1 ↓	skn-1 ↑	longevity ↑			
daf-18 ↓	c_PKB ↑	gsk-3 ↓	skn-1 ↑	longevity ↑		
ins-7 ↑	daf-2 ↑	ist-1 ↑	let-60 ↑	skn-1 ↑	longevity ↑	
daf-28 ↑	daf-2 ↑	ist-1 ↑	let-60 ↑	skn-1 ↑	longevity ↑	
ins-1 ↑	daf-2 ↑	ist-1 ↑	let-60 ↑	skn-1 ↑	longevity ↑	
ins-4 ↑	daf-2 ↑	ist-1 ↑	let-60 ↑	skn-1 ↑	longevity ↑	
ins-5 ↑	daf-2 ↑	ist-1 ↑	let-60 ↑	skn-1 ↑	longevity ↑	
ins-17 ↑	daf-2 ↑	ist-1 ↑	let-60 ↑	skn-1 ↑	longevity ↑	
ins-22 ↑	daf-2 ↑	ist-1 ↑	let-60 ↑	skn-1 ↑	longevity ↑	
ins-23 ↑	daf-2 ↑	ist-1 ↑	let-60 ↑	skn-1 ↑	longevity ↑	
ins-37 ↑	daf-2 ↑	ist-1 ↑	let-60 ↑	skn-1 ↑	longevity ↑	

Electronic Supplementary Material (ESI) for Molecular BioSystems
This journal is © The Royal Society of Chemistry 2013

Supplementary Figure 8 Secondary effect (decrease in longevity), N2i vs. *rheb-1*i

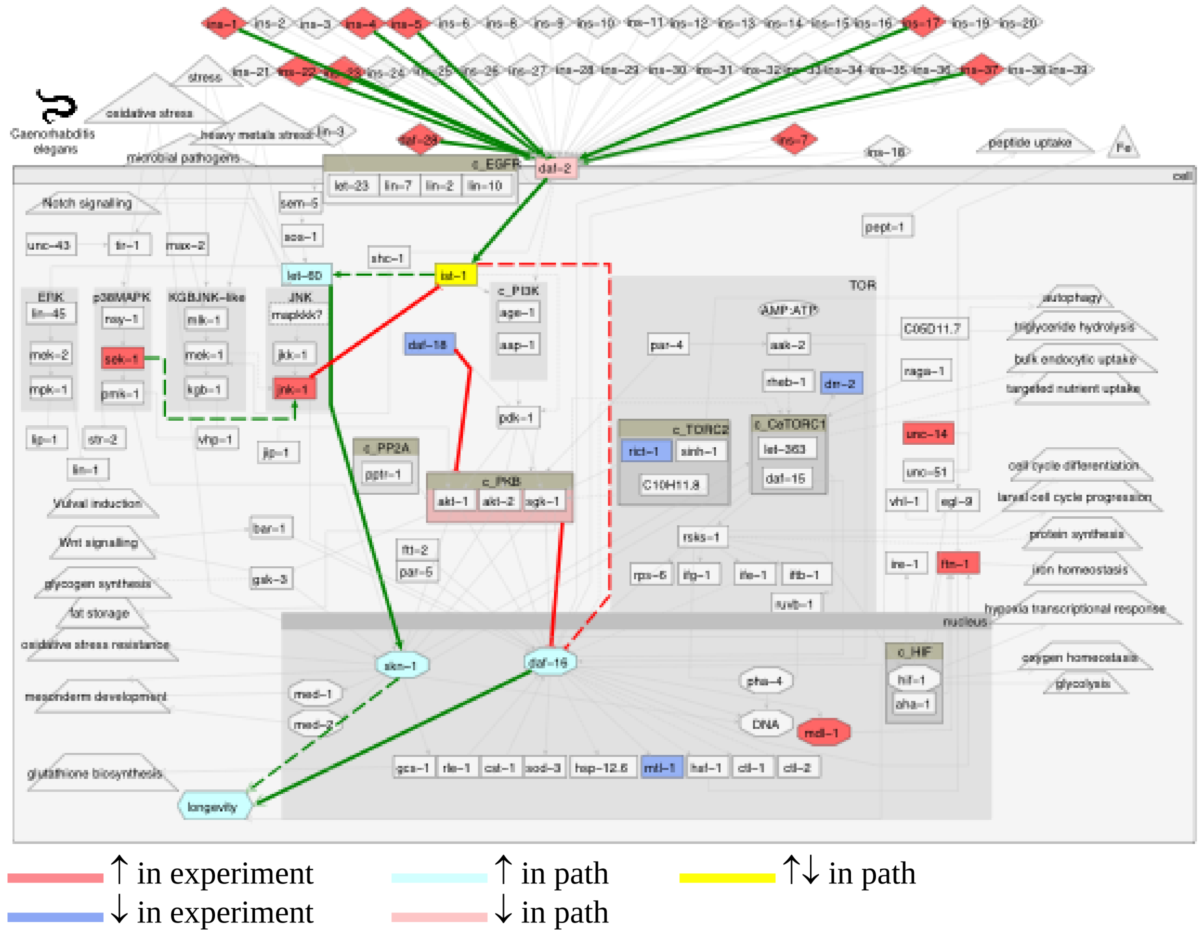

Secondary effects ↓Longevity						
daf-18 ↓	c_PKB↑	daf-16 ↓	longevity ↓			
jnk-1↑	ist-1 ↓	let-60 ↓	skn-1 ↓	longevity ↓		
ins-37↑	daf-2↑	ist-1↑	daf-16 ↓	longevity ↓		
daf-28↑	daf-2↑	ist-1↑	daf-16 ↓	longevity ↓		
ins-1↑	daf-2↑	ist-1↑	daf-16 ↓	longevity ↓		
ins-4↑	daf-2↑	ist-1↑	daf-16 ↓	longevity ↓		
ins-5↑	daf-2↑	ist-1↑	daf-16 ↓	longevity ↓		
ins-17↑	daf-2↑	ist-1↑	daf-16 ↓	longevity ↓		
ins-22↑	daf-2↑	ist-1↑	daf-16 ↓	longevity ↓		
ins-23↑	daf-2↑	ist-1↑	daf-16 ↓	longevity ↓		
sek-1↑	jnk-1↑	ist-1 ↓	let-60 ↓	skn-1 ↓	longevity ↓	

This journal is © The Royal Society of Chemistry 2013

Supplementary Figure 9 Primary effect (increase in longevity), N2i vs. *let-363*i

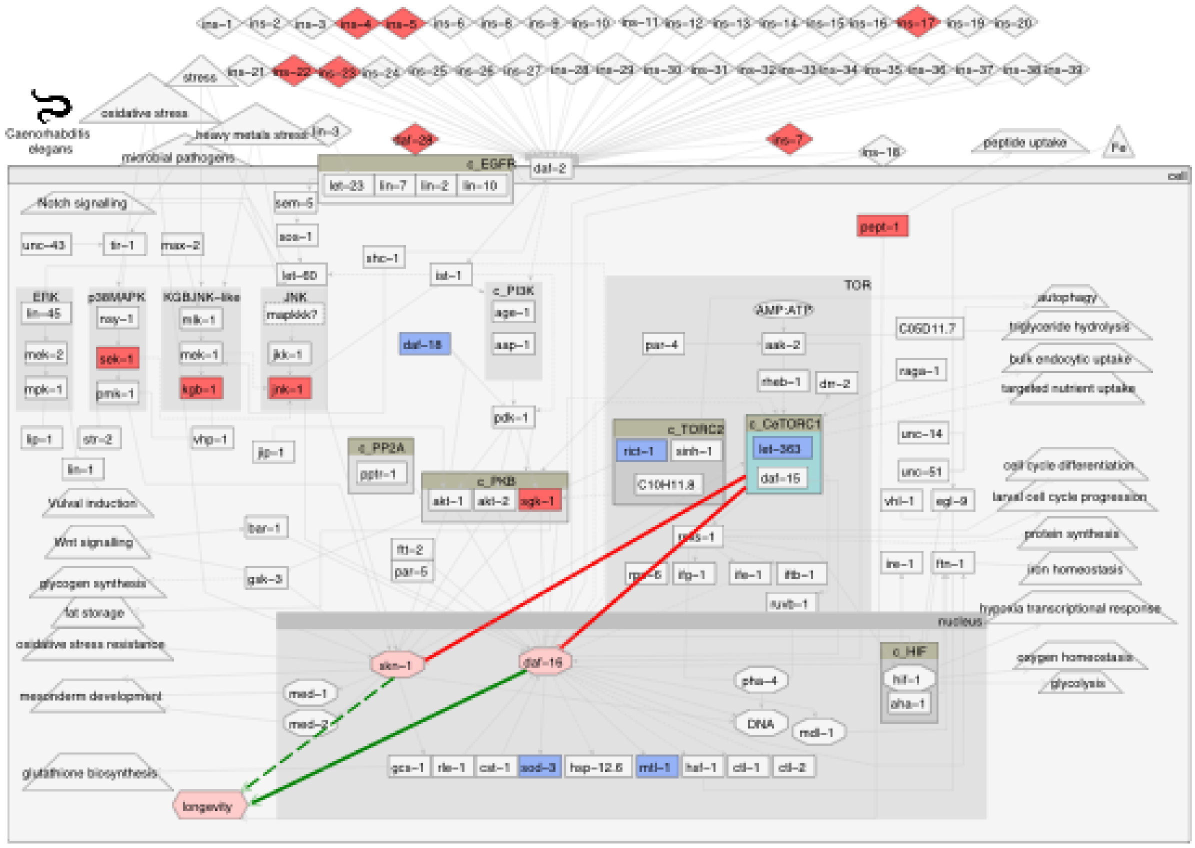

↑ in experiment ↑ in path ↑↓ in path
↓ in experiment ↓ in path

Primary effects ↑Longevity						
let-363 ↓	c_CeTORC1 ↓	skn-1 ↑	longevity ↑			
let-363 ↓	c_CeTORC1 ↓	daf-16 ↑	longevity ↑			

Electronic Supplementary Material (ESI) for Molecular BioSystems
This journal is © The Royal Society of Chemistry 2013

Supplementary Figure 10 Primary effect (decrease in longevity), N2i vs. *let-363*i

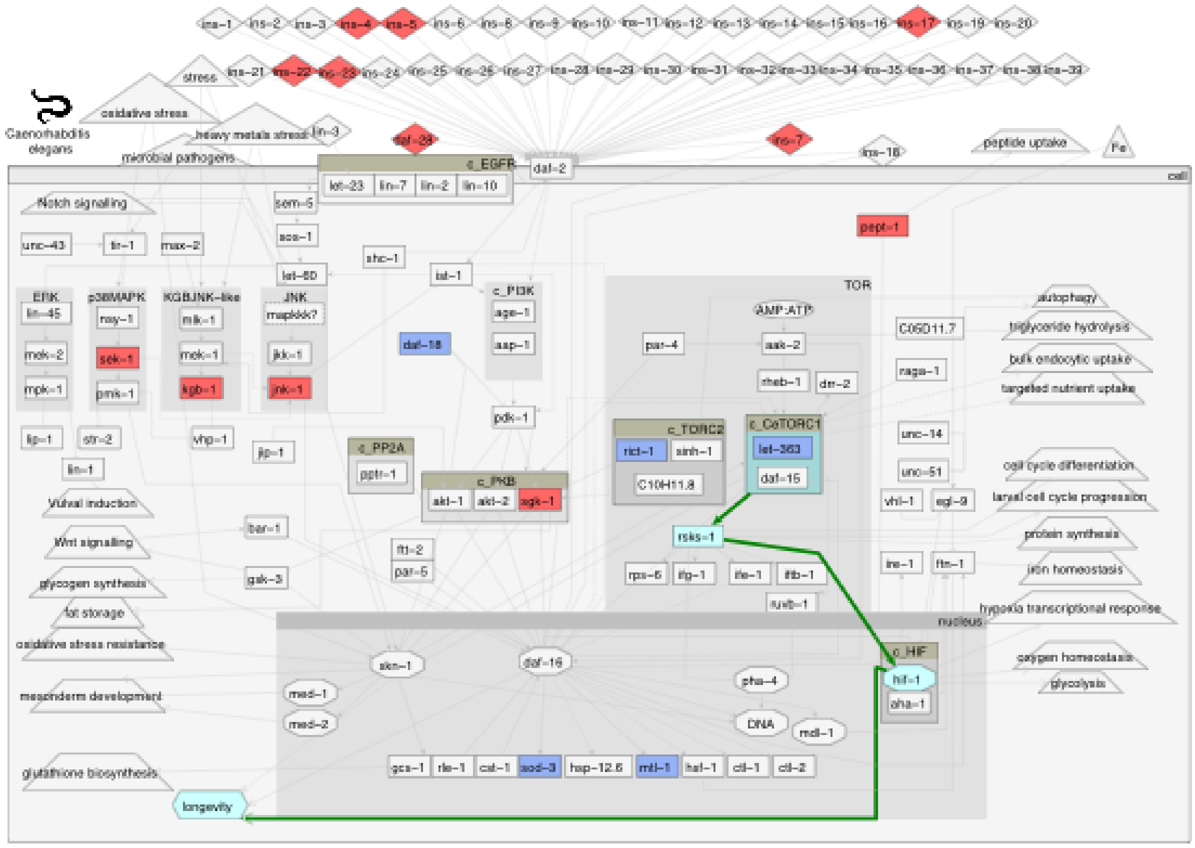

↑ in experiment ↓ in experiment ↑ in path ↓ in path ↑↓ in path

Primary effects ↓Longevity						
let-363 ↓	c_CeTORC1 ↓	rsks-1 ↓	hif-1 ↓	longevity ↓		

Electronic Supplementary Material (ESI) for Molecular BioSystems
This journal is © The Royal Society of Chemistry 2013

Supplementary Figure 11 Secondary effect (increase in longevity), N2i vs. *let-363*i

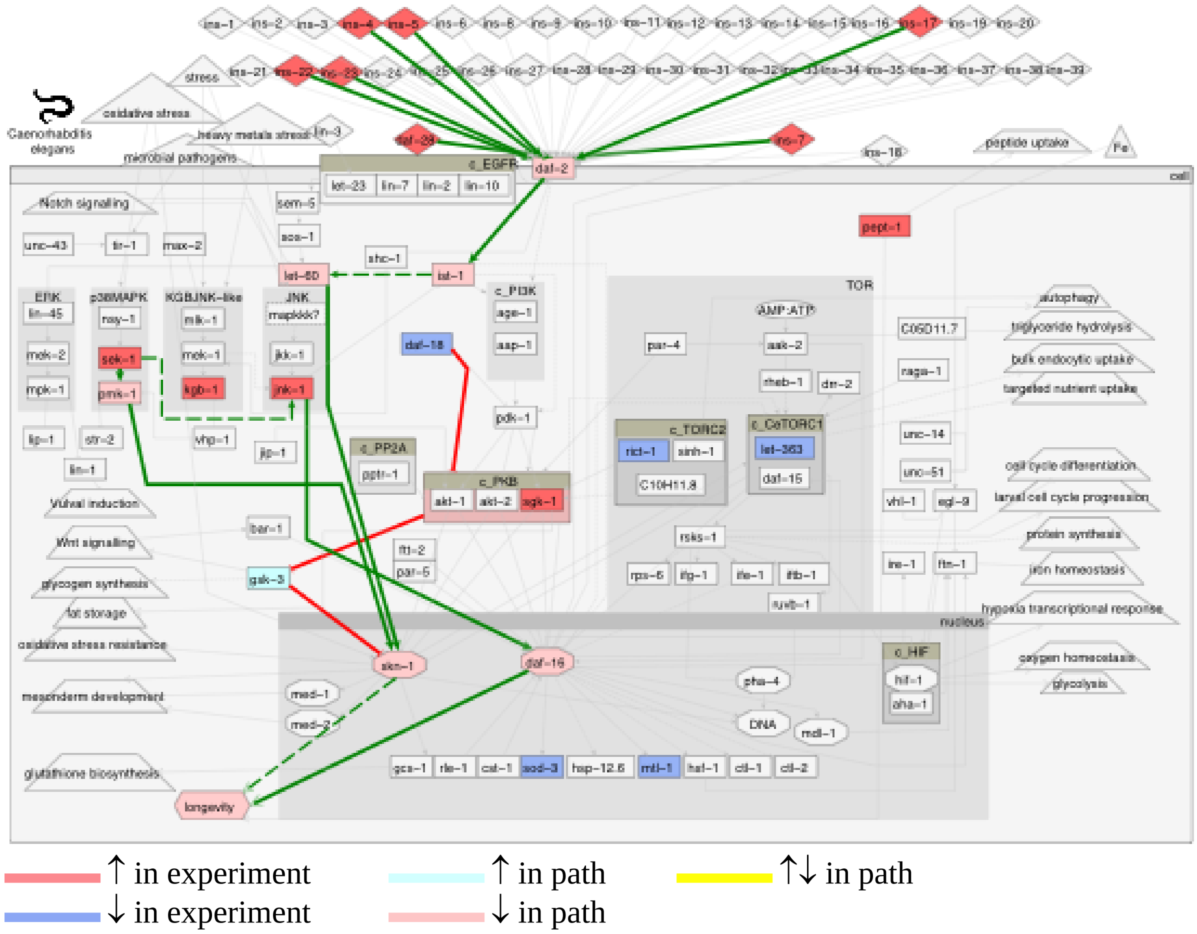

Secondary effects ↑Longevity						
jnk-1 ↑	daf-16 ↑	longevity ↑				
sek-1 ↑	jnk-1 ↑	daf-16 ↑	longevity ↑			
sek-1 ↑	pmk-1 ↑	skn-1 ↑	longevity ↑			
daf-18 ↓	c_PKB ↑	gsk-3 ↓	skn-1 ↑	longevity ↑		
ins-7 ↑	daf-2 ↑	ist-1 ↑	let-60 ↑	skn-1 ↑	longevity ↑	
daf-28 ↑	daf-2 ↑	ist-1 ↑	let-60 ↑	skn-1 ↑	longevity ↑	
ins-4 ↑	daf-2 ↑	ist-1 ↑	let-60 ↑	skn-1 ↑	longevity ↑	
ins-5 ↑	daf-2 ↑	ist-1 ↑	let-60 ↑	skn-1 ↑	longevity ↑	
ins-17 ↑	daf-2 ↑	ist-1 ↑	let-60 ↑	skn-1 ↑	longevity ↑	
ins-22 ↑	daf-2 ↑	ist-1 ↑	let-60 ↑	skn-1 ↑	longevity ↑	
ins-23 ↑	daf-2 ↑	ist-1 ↑	let-60 ↑	skn-1 ↑	longevity ↑	

Electronic Supplementary Material (ESI) for Molecular BioSystems
This journal is © The Royal Society of Chemistry 2013

Supplementary Figure 12 Secondary effect (decrease in longevity), N2i vs. *let-363*i

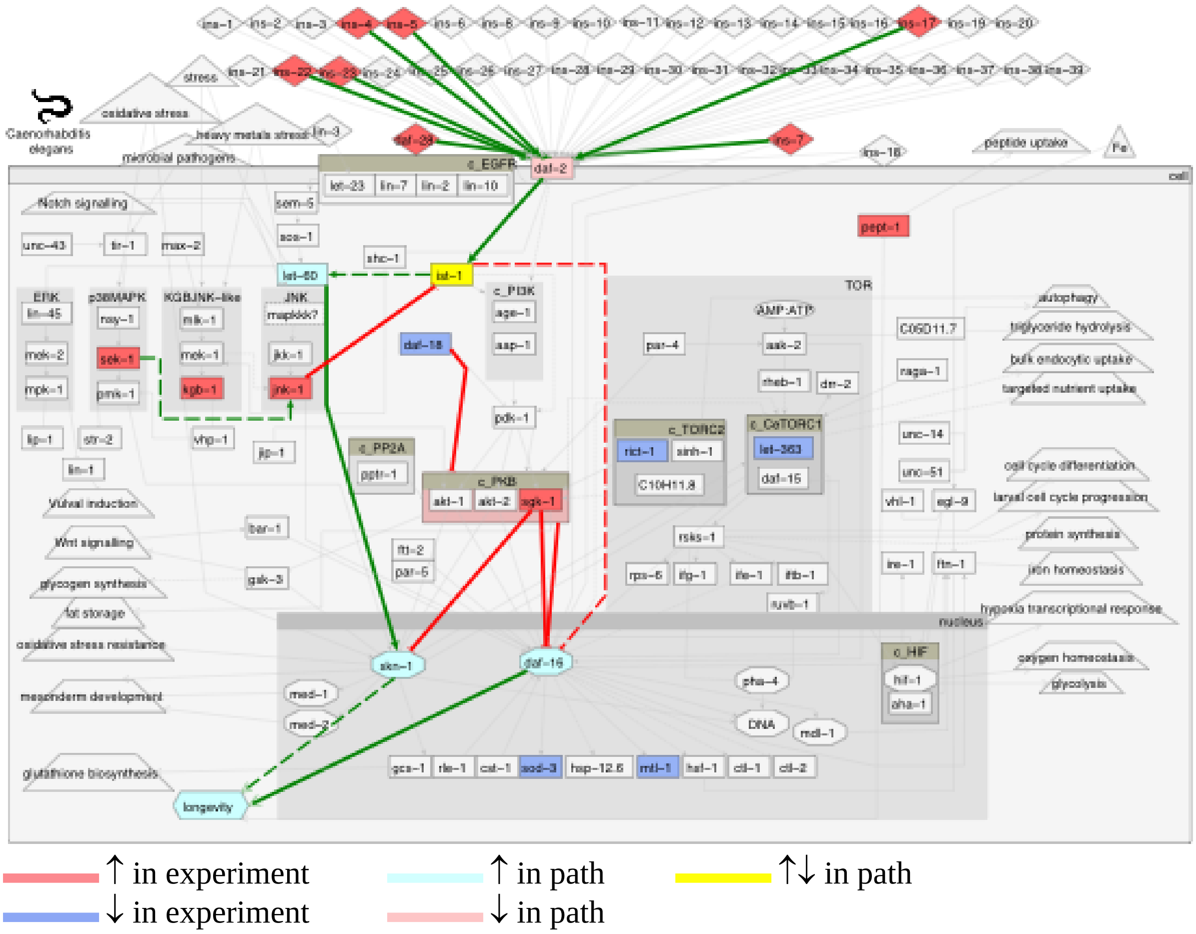

Secondary effects ↓Longevity						
sgk-1 ↑	daf-16 ↓	longevity ↓				
sgk-1 ↑	skn-1 ↓	longevity ↓				
daf-18 ↓	c_PKB ↑	daf-16 ↓	longevity ↓			
jnk-1 ↑	ist-1 ↓	let-60 ↓	skn-1 ↓	longevity ↓		
daf-28 ↑	daf-2 ↑	ist-1 ↑	daf-16 ↓	longevity ↓		
ins-7 ↑	daf-2 ↑	ist-1 ↑	daf-16 ↓	longevity ↓		
ins-4 ↑	daf-2 ↑	ist-1 ↑	daf-16 ↓	longevity ↓		
ins-5 ↑	daf-2 ↑	ist-1 ↑	daf-16 ↓	longevity ↓		
ins-17 ↑	daf-2 ↑	ist-1 ↑	daf-16 ↓	longevity ↓		
ins-22 ↑	daf-2 ↑	ist-1 ↑	daf-16 ↓	longevity ↓		
ins-23 ↑	daf-2 ↑	ist-1 ↑	daf-16 ↓	longevity ↓		
sek-1 ↑	jnk-1 ↑	ist-1 ↓	let-60 ↓	skn-1 ↓	longevity ↓	

This journal is © The Royal Society of Chemistry 2013

Supplementary Figure 13 Primary effect (increase in longevity), N2 vs. *aak-2* (over-expression)

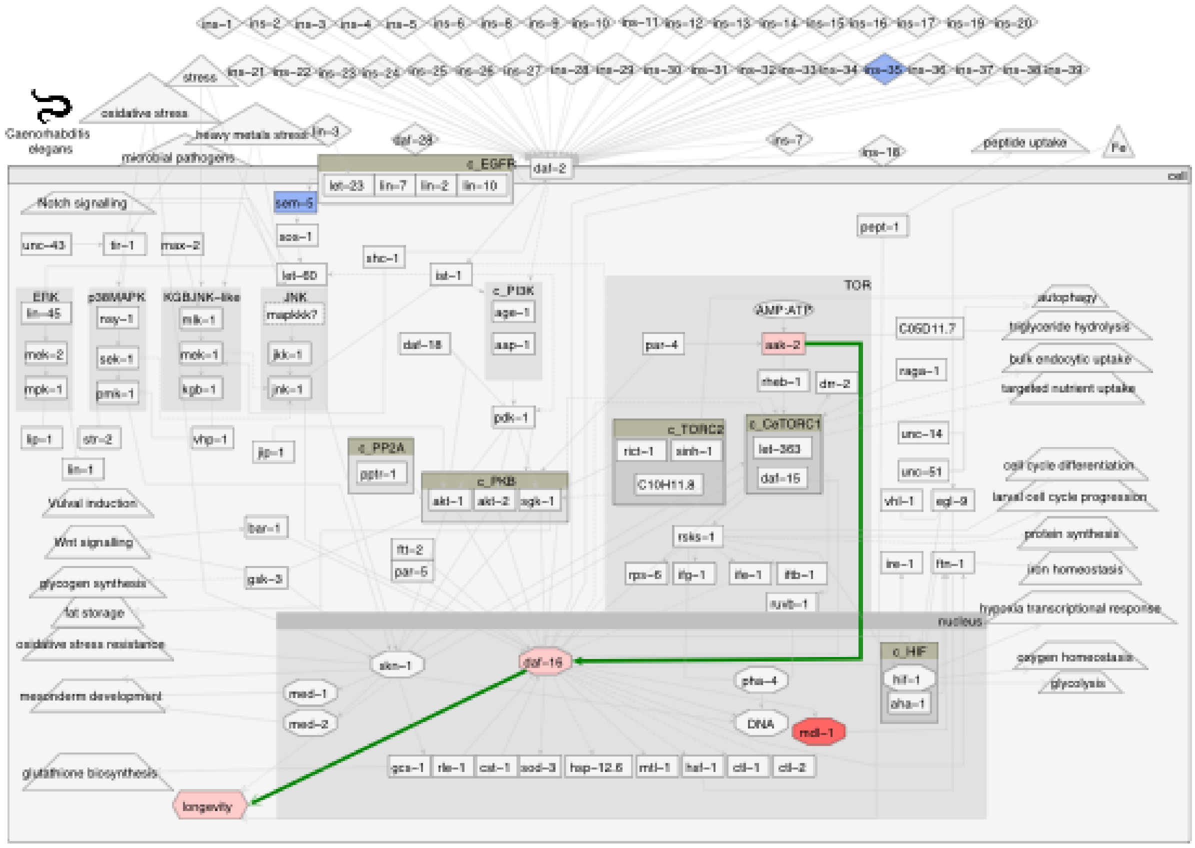

↑ in experiment	↑ in path
↓ in experiment	↓ in path
↑↓ in path	

Primary effects ↑Longevity						
aak-2 ↑	daf-16 ↑	longevity ↑				

Supplementary Figure 14 Primary effect (decrease in longevity), N2 vs. *aak-2* (over-expression)

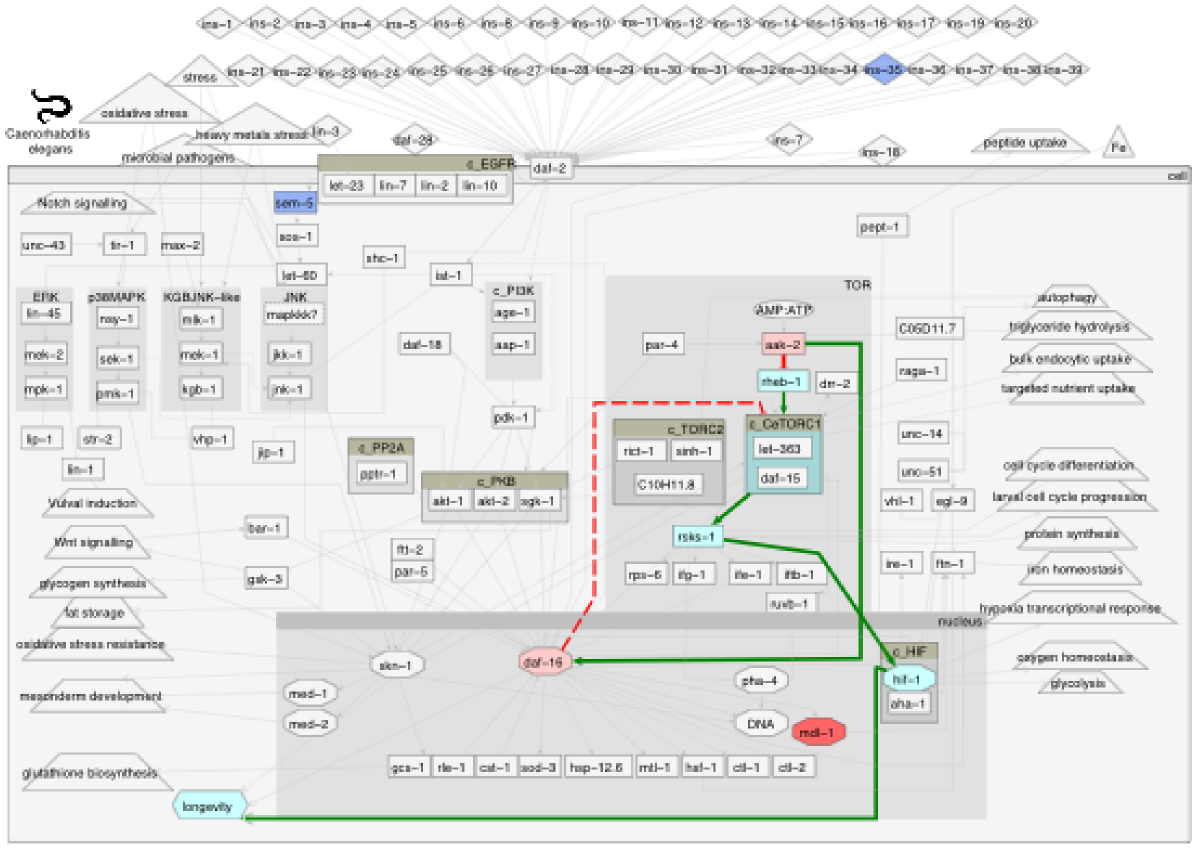

↑ in experiment
↓ in experiment
↑ in path
↓ in path
↑↓ in path

Primary effects ↓Longevity						
aak-2 ↑	daf-16 ↑	c_CeTORC1 ↓	rsks-1 ↓	hif-1 ↓	longevity ↓	
aak-2 ↑	rheb-1 ↓	c_CeTORC1 ↓	rsks-1 ↓	hif-1 ↓	longevity ↓	

Supplementary Figure 15 Secondary effect (increase in longevity), N2 vs. *aak-2* (over-expression)

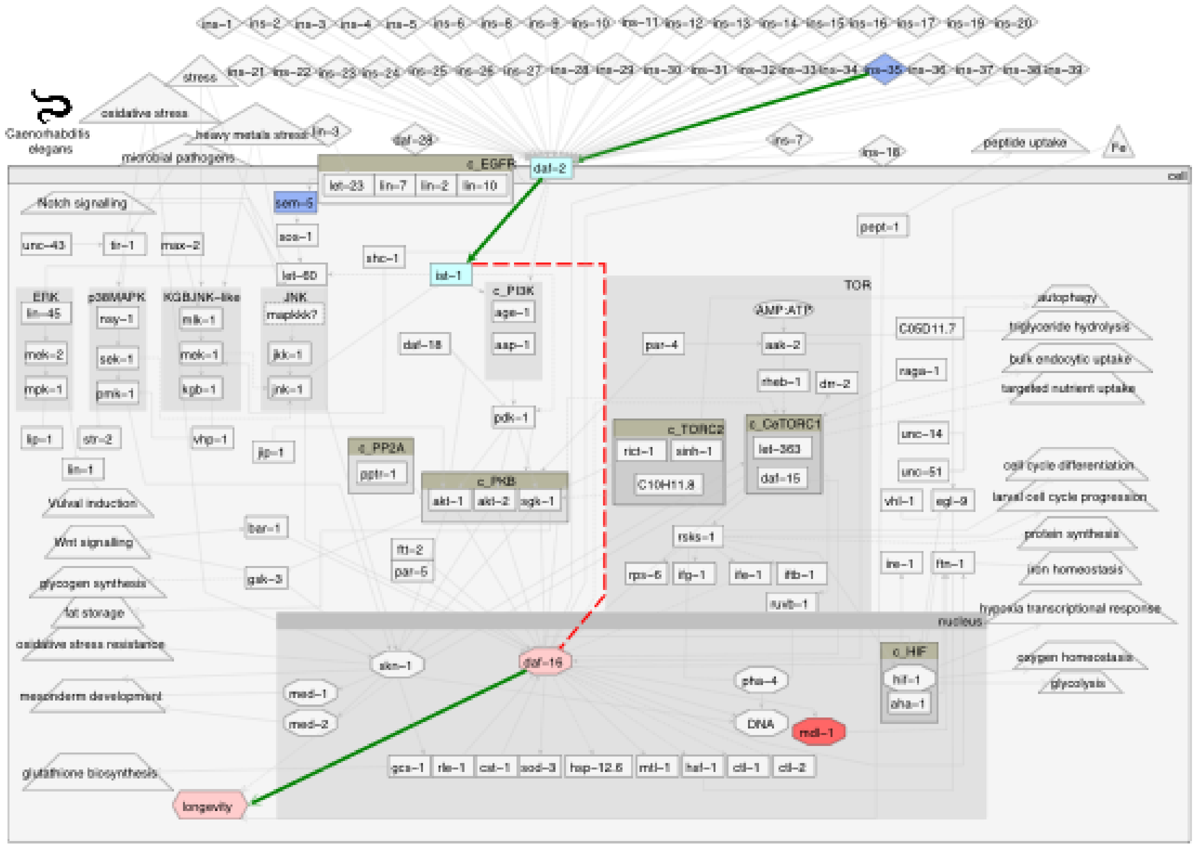

↑ in experiment ↑ in path ↑↓ in path
↓ in experiment ↓ in path

Secondary effects ↑Longevity						
ins-35 ↓	daf-2 ↓	ist-1 ↓	daf-16 ↑	longevity ↑	longevity ↓	

This journal is © The Royal Society of Chemistry 2013

Supplementary Figure 16 Secondary effect (decrease in longevity), N2 vs. *aak-2* (over-expression)

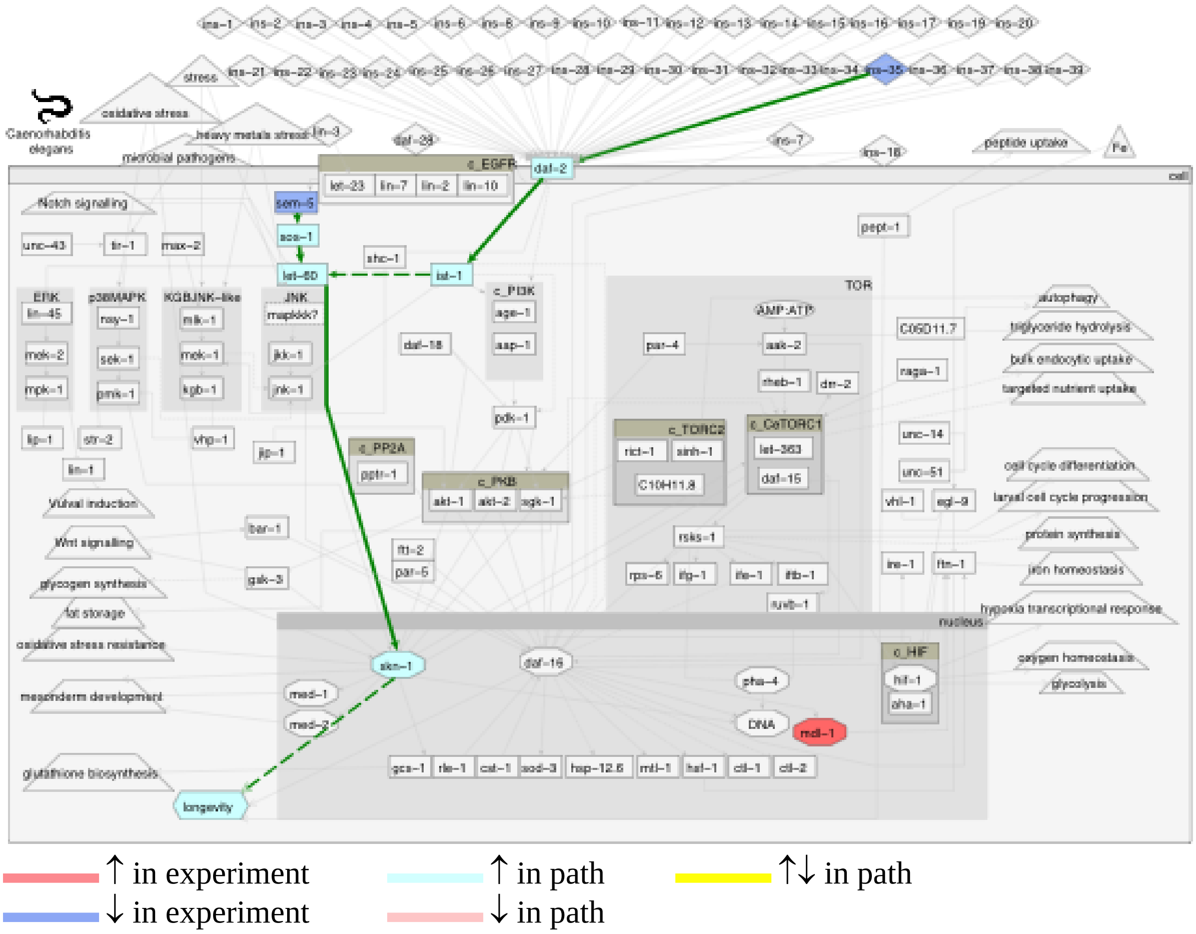

Secondary effects ↓Longevity						
sem-5 ↓	sos-1 ↓	let-60 ↓	skn-1 ↓	longevity ↓		
ins-35 ↓	daf-2 ↓	ist-1 ↓	let-60 ↓	skn-1 ↓	longevity ↓	

Electronic Supplementary Material (ESI) for Molecular BioSystems
This journal is © The Royal Society of Chemistry 2013

Supplementary Figure 17 Primary effect (decrease in longevity), *InR* vs. *InR;foxo*

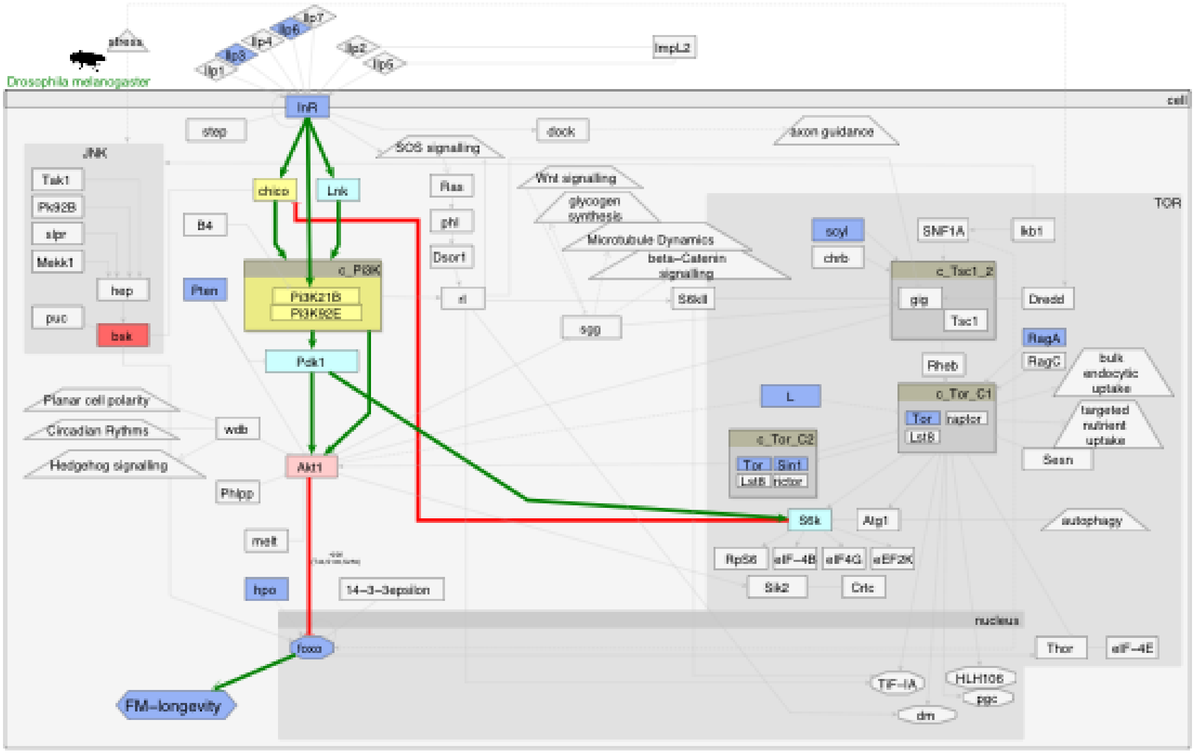

↑ in experiment ↑ in path ↑↓ in path
↓ in experiment ↓ in path

Primary effects ↓Longevity									
foxo ↓	longevity ↓								
InR ↓	Lnk↓	c_Pi3K ↓	Pdk1 ↓	S6k ↓	chico ↑	c_Pi3K ↑	Akt1 ↑	foxo ↓	longevity ↓
InR ↓	chico ↓	c_Pi3K ↓	Pdk1 ↓	S6k ↓	chico ↑	c_Pi3K ↑	Akt1 ↑	foxo ↓	longevity ↓

Electronic Supplementary Material (ESI) for Molecular BioSystems
This journal is © The Royal Society of Chemistry 2013

Supplementary Figure 18 Secondary effect (decrease in longevity), *InR* vs. *InR;foxo*

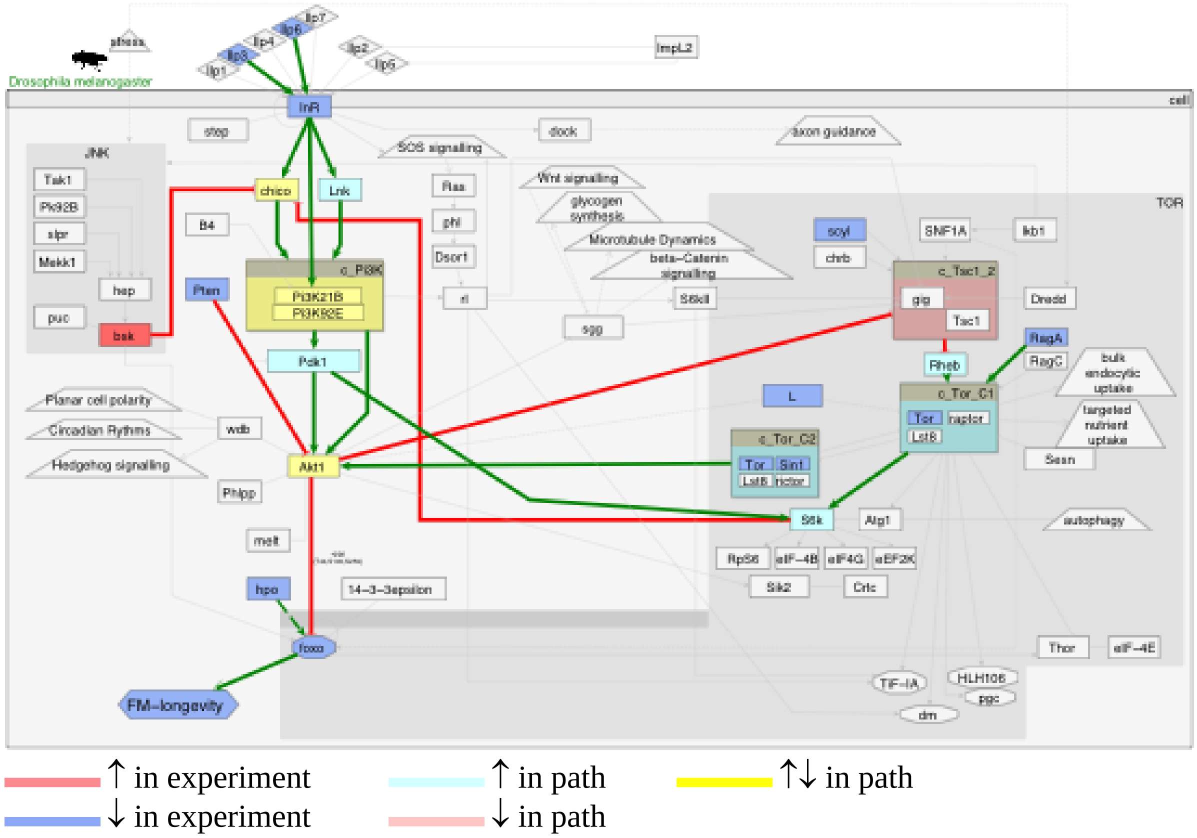

Primary effects ↓Longevity											
hpo ↓	foxo ↓	longevity ↓									
Pten ↓	Akt1 ↑	foxo ↓	longevity ↓								
RagA ↓	c_Tor_C1 ↓	S6k ↓	chico ↑	c_Pi3K ↑	Akt1 ↑	foxo ↓	longevity ↓				
Tor ↓	c_Tor_C1 ↓	S6k ↓	chico ↑	c_Pi3K ↑	Akt1 ↑	foxo ↓	longevity ↓				
bsk_dJNK ↑	chico ↓	c_Pi3K ↓	Pdk1 ↓	S6k ↓	chico ↑	c_Pi3K ↑	Akt1 ↑	foxo ↓	longevity ↓		
Ilp6 ↓	InR ↓	Lnk↓	c_Pi3K ↓	Pdk1 ↓	S6k ↓	chico ↑	c_Pi3K ↑	Akt1 ↑	foxo ↓	longevity ↓	
Ilp6 ↓	InR ↓	chico ↓	c_Pi3K ↓	Pdk1 ↓	S6k ↓	chico ↑	c_Pi3K ↑	Akt1 ↑	foxo ↓	longevity ↓	
Ilp3 ↓	InR ↓	Lnk↓	c_Pi3K ↓	Pdk1 ↓	S6k ↓	chico ↑	c_Pi3K ↑	Akt1 ↑	foxo ↓	longevity ↓	
Ilp3 ↓	InR ↓	chico ↓	c_Pi3K ↓	Pdk1 ↓	S6k ↓	chico ↑	c_Pi3K ↑	Akt1 ↑	foxo ↓	longevity ↓	
Sin1 ↓	c_Tor_C2 ↓	Akt1 ↓	c_Tsc1_2 ↑	Rheb ↓	c_Tor_C1 ↓	S6k ↓	chico ↑	c_Pi3K ↑	Akt1 ↑	foxo ↓	longevity ↓

Supplementary Table 1 List of gene names and ensembl identification data, part of IIS and TOR pathways

Gene Name	WormBase ID	Ensembl Gene ID
aak-2	WBGene00020142	T01C8.1
aap-1	WBGene00000001	Y110A7A.10
age-1	WBGene00000090	B0334.8
aha-1	WBGene00000095	C25A1.11
akt-1	WBGene00000102	C12D8.10
akt-2	WBGene00000103	F28H6.1
bar-1	WBGene00000238	C54D1.6
C05D11.7	WBGene00015484	C05D11.7
C10H11.8	WBGene00015697	C10H11.8
cst-1	WBGene00017472	F14H12.4
ctl-1	WBGene00000830	Y54G11A.6
ctl-2	WBGene00000831	Y54G11A.5
daf-15	WBGene00000911	C10C5.6
daf-16	WBGene00000912	R13H8.1
daf-18	WBGene00000913	T07A9.6
daf-2	WBGene00000898	Y55D5A.5
daf-28	WBGene00000920	Y116F11B.1
drr-2	WBGene00011730	T12D8.2
egl-9	WBGene00001178	F22E12.4
ftn-1	WBGene00001500	C54F6.14
ftt-2	WBGene00001502	F52D10.3
gcs-1	WBGene00001527	F37B12.2
gsk-3	WBGene00001746	Y18D10A.5
hif-1	WBGene00001851	F38A6.3
hsf-1	WBGene00002004	Y53C10A.12
hsp-12.6	WBGene00002013	F38E11.2
ife-1	WBGene00002059	F53A2.6
ifg-1	WBGene00002066	M110.4
iftb-1	WBGene00010560	K04G2.1
ins-1	WBGene00002084	F13B12.5
ins-10	WBGene00002093	T08G5.12
ins-11	WBGene00002094	C17C3.4
ins-12	WBGene00002095	C17C3.19
ins-13	WBGene00002096	C17C3.18
ins-14	WBGene00002097	F41G3.16
ins-15	WBGene00002098	F41G3.17
ins-16	WBGene00002099	Y39A3A.5
ins-17	WBGene00002100	F56F3.6
ins-18	WBGene00002101	T28B8.2
ins-19	WBGene00002102	T10D4.13
ins-2	WBGene00002085	ZK75.2
ins-20	WBGene00002103	ZK84.7
ins-21	WBGene00002104	M04D8.1
ins-22	WBGene00002105	M04D8.2
ins-23	WBGene00002106	M04D8.3
ins-24	WBGene00002107	ZC334.3
ins-25	WBGene00002108	ZC334.8
ins-26	WBGene00002109	ZC334.1
ins-27	WBGene00002110	ZC334.11
ins-28	WBGene00002111	ZC334.9
ins-29	WBGene00002112	ZC334.10
ins-3	WBGene00002086	ZK75.3
ins-30	WBGene00002113	ZC334.2
ins-31	WBGene00002114	T10D4.4
ins-32	WBGene00002115	Y8A9A.6
ins-33	WBGene00002116	W09C5.4
ins-34	WBGene00002117	F52B11.6
ins-35	WBGene00002118	K02E2.4
ins-36	WBGene00002119	Y53H1A.4
ins-37	WBGene00002120	F08G2.6
ins-38	WBGene00002121	C17C3.20
ins-39	WBGene00017668	F21E9.4
ins-4	WBGene00002087	ZK75.1
ins-5	WBGene00002088	ZK84.3
ins-6	WBGene00002089	ZK84.6

Electronic Supplementary Material (ESI) for Molecular BioSystems
This journal is © The Royal Society of Chemistry 2013

ins-7	WBGene00002090	ZK1251.2
ins-8	WBGene00002091	ZK1251.11
ins-9	WBGene00002092	C06E2.8
ire-1	WBGene00002147	C41C4.4
ist-1	WBGene00002163	C54D1.3
jip-1	WBGene00002176	F56D12.4
jkk-1	WBGene00002177	F35C8.3
jnk-1	WBGene00002178	B0478.1
kgb-1	WBGene00002187	T07A9.3
let-23	WBGene00002299	ZK1067.1
let-363	WBGene00002583	B0261.2
let-60	WBGene00002335	ZK792.6
lin-1	WBGene00002990	C37F5.1
lin-10	WBGene00002999	C09H6.2
lin-2	WBGene00002991	F17E5.1
lin-3	WBGene00002992	F36H1.4
lin-45	WBGene00003030	Y73B6A.5
lin-7	WBGene00002996	Y54G11A.10
lip-1	WBGene00003043	C05B10.1
max-2	WBGene00003144	Y38F1A.10
mdl-1	WBGene00003163	R03E9.1
med-1	WBGene00003180	T24D3.1
med-2	WBGene00003181	K04C2.6
mek-1	WBGene00003185	K08A8.1
mek-2	WBGene00003186	Y54E10BL.6
mlk-1	WBGene00003374	K11D12.10
mpk-1	WBGene00003401	F43C1.2
mtl-1	WBGene00003473	K11G9.6
nsy-1	WBGene00003822	F59A6.1
par-4	WBGene00003919	Y59A8B.14
par-5	WBGene00003920	M117.2
pdk-1	WBGene00003965	H42K12.1
pept-1	WBGene00003877	K04E7.2
pha-4	WBGene00004013	F38A6.1
pmk-1	WBGene00004055	B0218.3
pptr-1	WBGene00012348	W08G11.4
raga-1	WBGene00006414	T24F1.1
rheb-1	WBGene00010038	F54C8.5
rict-1	WBGene00009245	F29C12.3
rle-1	WBGene00010923	M142.6
rps-6	WBGene00004475	Y71A12B.1
rsks-1	WBGene00012929	Y47D3A.16
ruvb-1	WBGene00007784	C27H6.2
sek-1	WBGene00004758	R03G5.2
sem-5	WBGene00004774	C14F5.5
sgk-1	WBGene00004789	W10G6.2
shc-1	WBGene00018788	F54A5.3
sinh-1	WBGene00013261	Y57A10A.20
skn-1	WBGene00004804	T19E7.2
sod-3	WBGene00004932	C08A9.1
sos-1	WBGene00004947	T28F12.3
str-2	WBGene00006070	C50C10.7
tir-1	WBGene00006575	F13B10.1
unc-14	WBGene00006753	K10D3.2
unc-43	WBGene00006779	K11E8.1
unc-51	WBGene00006786	Y60A3A.1
vhl-1	WBGene00006922	F08G12.4
vhp-1	WBGene00006923	F08B1.1

Supplementary Table 2 List of references used in generating the *Caenorhabditis elegans* IIS and TOR pathways

1. Y. Matsunaga, K. Gengyo-Ando, S. Mitani, T. Iwasaki and T. Kawano, *Biochem Biophys Res Commun*, 2012, **423**, 478-483.
2. S. Robida-Stubbs, K. Glover-Cutter, D. W. Lamming, M. Mizunuma, S. D. Narasimhan, E. Neumann-Haefelin, D. M. Sabatini and T. K. Blackwell, *Cell Metab*, 2012, **15**, 713-724.
3. D. Ackerman and D. Gems, *PLoS Genet*, 2012, **8**, e1002498.
4. D. Z. Korta, S. Tuck and E. J. Hubbard, *Development*, 2012, **139**, 859-870.
5. R. Loewith and M. N. Hall, *Genetics*, 2011, **189**, 1177-1201.
6. C. Rongo, *Aging (Albany NY)*, 2011, **3**, 896-905.
7. G. Liu, J. Rogers, C. T. Murphy and C. Rongo, *Embo J*, 2011, **30**, 2990-3003.
8. S. F. Leiser, A. Begun and M. Kaeberlein, *Aging Cell*, 2011, **10**, 318-326.
9. T. Okuyama, H. Inoue, S. Ookuma, T. Satoh, K. Kano, S. Honjoh, N. Hisamoto, K. Matsumoto and E. Nishida, *J Biol Chem*, 2010, **285**, 30274-30281.
10. M. A. Schreiber, J. T. Pierce-Shimomura, S. Chan, D. Parry and S. L. McIntire, *PLoS Genet*, 2010, **6**, e1000972.
11. T. T. Ching, A. B. Paal, A. Mehta, L. Zhong and A. L. Hsu, *Aging Cell*, 2010, **9**, 545-557.
12. B. Lant and K. B. Storey, *Int J Biol Sci*, 2010, **6**, 9-50.
13. K. Fujiki, T. Mizuno, N. Hisamoto and K. Matsumoto, *Mol Cell Biol*, 2010, **30**, 995-1003.
14. D. Chen, E. L. Thomas and P. Kapahi, *PLoS Genet*, 2009, **5**, e1000486.
15. K. T. Jones, E. R. Greer, D. Pearce and K. Ashrafi, *PLoS Biol*, 2009, **7**, e60.
16. S. Padmanabhan, A. Mukhopadhyay, S. D. Narasimhan, G. Tesz, M. P. Czech and H. A. Tissenbaum, *Cell*, 2009, **136**, 939-951.
17. A. A. Soukas, E. A. Kane, C. E. Carr, J. A. Melo and G. Ruvkun, *Genes Dev*, 2009, **23**, 496-511.
18. S. Honjoh, T. Yamamoto, M. Uno and E. Nishida, *Nature*, 2009, **457**, 726-730.
19. P. Narbonne and R. Roy, *Nature*, 2009, **457**, 210-214.
20. E. Neumann-Haefelin, W. Qi, E. Finkbeiner, G. Walz, R. Baumeister and M. Hertweck, *Genes Dev*, 2008, **22**, 2721-2735.
21. T. Mizuno, K. Fujiki, A. Sasakawa, N. Hisamoto and K. Matsumoto, *Mol Cell Biol*, 2008, **28**, 7041-7049.
22. K. L. Sheaffer, D. L. Updike and S. E. Mango, *Curr Biol*, 2008, **18**, 1355-1364.
23. H. Lee, J. S. Cho, N. Lambacher, J. Lee, S. J. Lee, T. H. Lee, A. Gartner and H. S. Koo, *J Biol Chem*, 2008, **283**, 14988-14993.
24. J. M. Tullet, M. Hertweck, J. H. An, J. Baker, J. Y. Hwang, S. Liu, R. P. Oliveira, R. Baumeister and T. K. Blackwell, *Cell*, 2008, **132**, 1025-1038.
25. Y. C. Kuo, K. Y. Huang, C. H. Yang, Y. S. Yang, W. Y. Lee and C. W. Chiang, *J Biol Chem*, 2008, **283**, 1882-1892.
26. E. L. Greer, D. Dowlatshahi, M. R. Banko, J. Villen, K. Hoang, D. Blanchard, S. P. Gygi and A. Brunet, *Curr Biol*, 2007, **17**, 1646-1656.
27. P. T. Bhaskar and N. Hay, *Dev Cell*, 2007, **12**, 487-502.
28. W. Li, B. Gao, S. M. Lee, K. Bennett and D. Fang, *Dev Cell*, 2007, **12**, 235-246.
29. K. Z. Pan, J. E. Palter, A. N. Rogers, A. Olsen, D. Chen, G. J. Lithgow and P. Kapahi, *Aging Cell*, 2007, **6**, 111-119.

30. M. Hansen, S. Taubert, D. Crawford, N. Libina, S. J. Lee and C. Kenyon, *Aging Cell*, 2007, **6**, 95-110.
31. K. Ogura and Y. Goshima, *Development*, 2006, **133**, 3441-3450.
32. Y. Wang, S. W. Oh, B. Deplancke, J. Luo, A. J. Walhout and H. A. Tissenbaum, *Mech Ageing Dev*, 2006, **127**, 741-747.
33. M. K. Lehtinen, Z. Yuan, P. R. Boag, Y. Yang, J. Villen, E. B. Becker, S. DiBacco, N. de la Iglesia, S. Gygi, T. K. Blackwell and A. Bonni, *Cell*, 2006, **125**, 987-1001.
34. J. H. An, K. Vranas, M. Lucke, H. Inoue, N. Hisamoto, K. Matsumoto and T. K. Blackwell, *Proc Natl Acad Sci U S A*, 2005, **102**, 16275-16280.
35. H. Inoue, N. Hisamoto, J. H. An, R. P. Oliveira, E. Nishida, T. K. Blackwell and K. Matsumoto, *Genes Dev*, 2005, **19**, 2278-2283.
36. M. V. Sundaram, *Genes Dev*, 2005, **19**, 1825-1839.
37. M. A. Essers, L. M. de Vries-Smits, N. Barker, P. E. Polderman, B. M. Burgering and H. C. Korswagen, *Science*, 2005, **308**, 1181-1184.
38. S. W. Oh, A. Mukhopadhyay, N. Svrzikapa, F. Jiang, R. J. Davis and H. A. Tissenbaum, *Proc Natl Acad Sci U S A*, 2005, **102**, 4494-4499.
39. K. Jia, D. Chen and D. L. Riddle, *Development*, 2004, **131**, 3897-3906.
40. B. Meissner, M. Boll, H. Daniel and R. Baumeister, *J Biol Chem*, 2004, **279**, 36739-36745.
41. T. Mizuno, N. Hisamoto, T. Terada, T. Kondo, M. Adachi, E. Nishida, D. H. Kim, F. M. Ausubel and K. Matsumoto, *Embo J*, 2004, **23**, 2226-2234.
42. M. Hertweck, C. Gobel and R. Baumeister, *Dev Cell*, 2004, **6**, 577-588.
43. J. H. An and T. K. Blackwell, *Genes Dev*, 2003, **17**, 1882-1893.
44. C. T. Murphy, S. A. McCarroll, C. I. Bargmann, A. Fraser, R. S. Kamath, J. Ahringer, H. Li and C. Kenyon, *Nature*, 2003, **424**, 277-283.
45. A. H. Kim, T. Sasaki and M. V. Chao, *J Biol Chem*, 2003, **278**, 29830-29836.
46. W. Li, S. G. Kennedy and G. Ruvkun, *Genes Dev*, 2003, **17**, 844-858.
47. N. Moghal and P. W. Sternberg, *Exp Cell Res*, 2003, **284**, 150-159.
48. C. A. Wolkow, M. J. Munoz, D. L. Riddle and G. Ruvkun, *J Biol Chem*, 2002, **277**, 49591-49597.
49. A. Villanueva, J. Lozano, A. Morales, X. Lin, X. Deng, M. O. Hengartner and R. N. Kolesnick, *Embo J*, 2001, **20**, 5114-5128.
50. M. F. Maduro, M. D. Meneghini, B. Bowerman, G. Broitman-Maduro and J. H. Rothman, *Mol Cell*, 2001, **7**, 475-485.
51. A. Sagasti, N. Hisamoto, J. Hyodo, M. Tanaka-Hino, K. Matsumoto and C. I. Bargmann, *Cell*, 2001, **105**, 221-232.
52. S. B. Pierce, M. Costa, R. Wisotzkey, S. Devadhar, S. A. Homburger, A. R. Buchman, K. C. Ferguson, J. Heller, D. M. Platt, A. A. Pasquinelli, L. X. Liu, S. K. Doberstein and G. Ruvkun, *Genes Dev*, 2001, **15**, 672-686.
53. A. Brunet, J. Park, H. Tran, L. S. Hu, B. A. Hemmings and M. E. Greenberg, *Mol Cell Biol*, 2001, **21**, 952-965.
54. S. Ogg and G. Ruvkun, *Mol Cell*, 1998, **2**, 887-893.
55. S. M. Kaech, C. W. Whitfield and S. K. Kim, *Cell*, 1998, **94**, 761-771.
56. J. N. Maloof and C. Kenyon, *Development*, 1998, **125**, 181-190.
57. Y. Wu, M. Han and K. L. Guan, *Genes Dev*, 1995, **9**, 742-755.
58. M. Han, A. Golden, Y. Han and P. W. Sternberg, *Nature*, 1993, **363**, 133-140.
